# Cellulose-Based Ion Exchange Membranes for Electrochemical Energy Systems: A Review

**DOI:** 10.3390/membranes15100304

**Published:** 2025-10-06

**Authors:** Nur Syahirah Faiha Shawalludin, Saidatul Sophia Sha’rani, Mohamed Azlan Suhot, Shamsul Sarip, Mohamed Mahmoud Nasef

**Affiliations:** 1 Advanced Materials Research Group, Centre of Hydrogen Energy, Universiti Teknologi Malaysia, Kuala Lumpur 54100, Malaysia; nursyahirahfaiha@graduate.utm.my (N.S.F.S.); saidatulsophia@utm.my (S.S.S.); 2 Razak Faculty of Technology and Informatics, Universiti Teknologi Malaysia, Kuala Lumpur 54100, Malaysia; 3 Faculty of Artificial Intelligence, Universiti Teknologi Malaysia, Kuala Lumpur 54100, Malaysia; azlans.kl@utm.my (M.A.S.); shamsuls.kl@utm.my (S.S.); 4 Department of Chemical and Environmental Engineering, Malaysia-Japan International Institute of Technology (MJIIT), Universiti Teknologi Malaysia, Kuala Lumpur 54000, Malaysia

**Keywords:** nanocellulose, cellulose-based membranes, electrochemical energy conversion and storage systems, fuel cells, batteries, supercapacitors, reverse electrodialysis

## Abstract

Cellulose, the most abundant polysaccharide on earth, possesses desirable properties such as biodegradability, low cost, and low toxicity, making it suitable for a wide range of applications. Being a non-conductive material, the structure of the nanocellulose can be modified or incorporated with conductive filler to facilitate charge transport between the polymer matrix and conductive components. Recently, cellulose-based ion exchange membranes (IEMs) have gained strong attention as alternatives to environmentally burdening synthetic polymers in electrochemical energy systems, owing to their renewable nature and versatile chemical structure. This article provides a comprehensive review of the structures, fabrication aspects and properties of various cellulose-based membranes for fuel cells and water electrolyzers, batteries, supercapacitors, and reverse electrodialysis (RED) applications. The scope includes an overview of various cellulose-based membrane fabrication methods, different forms of cellulose, and their applications in energy conversion and energy storage systems. The review also discusses the fundamentals of electrochemical energy systems, the role of IEMs, and recent advancements in the cellulose-based membranes’ research and development. Finally, it highlights current challenges to their performance and sustainability, along with recommendations for future research directions.

## 1. Introduction

The continuous progress of electrochemical technology is crucial in the continued quest for sustainable energy solutions. These innovative technologies play a fundamental role in the sustainable energy sector by enabling the conversion and efficient storage of energy, as seen in many applications such as fuel cells, batteries, water electrolyzers, and supercapacitors [1,2,3]. A common feature among these technologies is the use of ion exchange membranes (IEMs), which have undergone significant advancements over the past few decades. The intrinsic link between electrochemical technologies and the development of these membranes demonstrates their synergistic progress in the direction of sustainable and efficient energy systems [4].

IEMs are materials in the form of ion-conducting polymer films or sheets comprising polymer substrates with multi-functional or ion-exchange groups, enabling them to play a fundamental role in a wide range of electrochemical systems, including energy conversion and energy storage [5]. This role is not only limited to selective ion transport but also serves as a barrier separating the electrodes and the reactants [6]. Thus, IEMs are crucial components for maintaining charge balance, efficient reactions, and safe operation of electrochemical systems [7].

Based on the fixed functional groups, IEMs can be classified into three main classes, each tailored for conducting specific ions and operating conditions. These include cation/proton exchange membranes, anion exchange membranes, and bi-polar/combined membranes [5]. The IEMs can be further classified into homogeneous and heterogeneous membranes. Homogeneous membranes exhibit a uniform charge distribution, whereas heterogeneous membranes have a non-uniform charge distribution, caused by uncharged polymers separating the charged polymer domains [8]. Thus, the chemical and physical characteristics significantly influence the performance of IEMs.

The type of functional group in IEMs dictates the electrochemical applications. For instance, proton exchange membranes (PEMs), which conduct protons (H^+^), are used in proton exchange membrane fuel cells (PEMFCs) and microbial fuel cells (MFCs) [9], direct methanol fuel cells (DMFCs) [10], proton exchange membrane water electrolyzers (PEMWEs) [11], and electrochemical hydrogen compressors and pumps, which compress or purify hydrogen without mechanical components. PEMs have also been recently tested in a CO_2_ electrolyzer for converting CO_2_ into value-added chemicals in [12], as well as in some vanadium redox flow batteries (VRFBs) to eliminate vanadium crossover and transport protons [13]. On the other hand, anion exchange membranes (AEMs) conduct anions such as OH^−^, Cl^−^, and HCO_3_^−^ and are used in alkaline electrochemical systems, including anion exchange membrane fuel cells (AEMFCs), AEM water electrolyzers (AEMWEs), reverse electrodialysis (RED), CO_2_ electrolyzers in basic media, and electrochemical ammonia synthesis cells. AEMs allow operation in basic media and enable the use of non-precious metal catalysts, while also providing greater chemical tolerance for electrochemical systems [14]. Furthermore, AEMs were also used in flow batteries such as VRFBs and solid-state batteries, such as zinc–air, zinc–nickel, zinc–manganese, and some hybrid lithium– or sodium–air batteries.

Currently, commercial PEMs are mainly made of synthetic polymers such as perfluorosulfonic acid (PFSA) polymers (e.g., Nafion^®^, Aquivion^®^, and Flemion^®^), sulfonated poly(ether ether ketone) (SPEEK), and phosphoric acid-doped polybenzimidazole (PBI) [15]. Similarly, commercial AEMs such as FAA3-50, Sustainion, Aemion™, XION Composite, and PiperION™, as well as counterparts including quaternized poly(phenylene oxide), poly(ether sulfone), poly(vinylbenzyl chloride) functionalized with quaternary ammonium (−NR_3_^+^), and imidazolium- or piperidinium-functionalized poly(arylene)s, are also fabricated based on synthetic polymers [16]. Practically, PFSA PEMs have been widely used in industry and proven to have high proton conductivity coupled with excellent chemical stability and mechanical strength for low-temperature operations (<100 °C) [17]. However, the fabrication of these membranes is associated with adverse environmental impacts due to the reliance on traditional petroleum-based polymers and the use of hazardous fluorine chemistry. Therefore, there is a rising need to find alternative, environmentally friendly, and sustainable materials for such membranes.

The quest for the development of alternative PEMs and AEMs has turned to the exploration of a wide variety of natural materials to meet the pressing need for sustainable alternatives. Biopolymers, such as collagen, keratin, silk, polysaccharides, chitin, chitosan, silk fibroin and cellulose have shown great promise in this regard [6]. Derived from natural sources, these materials provide balance, practicality, and environmental sustainability and have been utilized in the development of a wide range of functional polymeric composites [18,19].

Cellulose, as the predominant source of biopolymer materials globally, has a significantly higher production rate compared to other natural sources [20]. Cellulose is found in plant cell walls and is gaining significant attention due to its abundance, renewable nature, and biodegradability. This is attributed to its linear composition consisting of repetitive glucose units, which are linked by β-1,4-glycosidic bonds, with many hydroxyl groups and distinctive characteristics, such as reasonable mechanical strength, chemical resilience, ease of functionalization, and water-adsorption capability. Despite these advantages, cellulose is intrinsically non-conductive in nature as it does not have functional groups that can easily facilitate anion or cation transport; hence, its direct application is limited as it cannot provide a proper conductive medium. To overcome this limitation, cellulose can be modified or incorporated with a conductive filler [21]. The versatility of cellulose stems from the ability to undergo diverse modifications using chemical and physical treatments. Such modifications allow for tuning its adsorption, dispersion of mechanical properties, and surface charge, thereby enhancing its effectiveness in specific applications. This versatility has facilitated the utilization of cellulose and its modified forms in diverse fields, including bioplastics, food packaging, water filtration, textiles, pharmaceuticals, food, and paper industries [22]. In addition, its intramolecular and intermolecular chemical groups related to its specific properties, such as dense packing and hydrophilicity, also made cellulose very appealing for applications in developing membranes [23]. More details on cellulose sources, forms, processing, and applications can be found in various published reviews [24,25,26].

**Figure 1:**
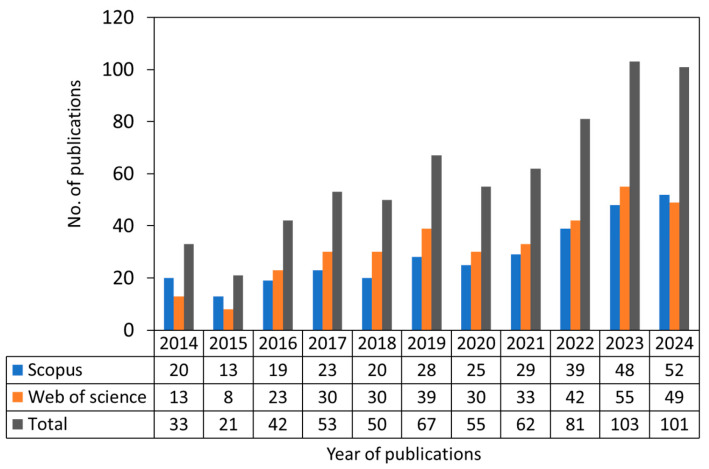
Publications in the past 10 years on cellulose-based membranes for electrochemical systems (database extracted from Scopus and Web of Science as of 5 June 2025).

Cellulose-based membranes have found widespread applications in various fields, including separation and purification, due to their renewable origin, offering a sustainable alternative material to synthetic polymers and contributing to the advancement of sustainable membrane technologies [27]. Particularly, cellulose-based membranes have found their use in water treatment (reverse osmosis, microfiltration, and nanofiltration), protein absorption, hemodialysis, catalysis, pervaporation, antifouling, and water treatment applications [28,29,30]. More details on the applications of cellulose-based membranes for separation applications can be found in a recent article published elsewhere [31]. Cellulose-based membranes have also found applications in electrochemical systems for generating electricity and energy storage. Thus, they have been the subject of numerous investigations, as indicated by the significant increase in published articles over the past decade. Figure 1 shows publications on cellulose-based membranes for electrochemical systems in the past 10 years. As an example, cellulose was used for fabricating separators for batteries such as lithium-ion batteries (LIBs) and lithium-sulfur batteries (LSBs) [1,26], capacitors such as supercapacitors and electric double-layer capacitors [2], and fuel cells [3,18]. Other innovative applications of cellulose-based membranes include their use in electrochromic displays [32] and osmotic energy and salinity harvesting [29,33].

Cellulose-based membranes for separation and purification applications have been reviewed on several occasions [27,28,31,34]. A review on advances in the fields of flexible wearable electronics, thermoelectric nanogenerators, mechanical energy nanogenerators, sensors, electrodes, and photovoltaic solar cells was also recently published [35]. Meanwhile, there have been several reviews on cellulose-based membranes for energy applications, primarily focused on specific applications such as LIBs [36], supercapacitors [37], and fuel cells [38]. Specific membranes made of carboxymethyl cellulose (CMC) were also recently reviewed [39]. However, a review article discussing the progress on the application of cellulose-based IEMs and separators on a broad basis concerning various electrochemical systems for renewable energy applications is lacking.

**Figure 2:**
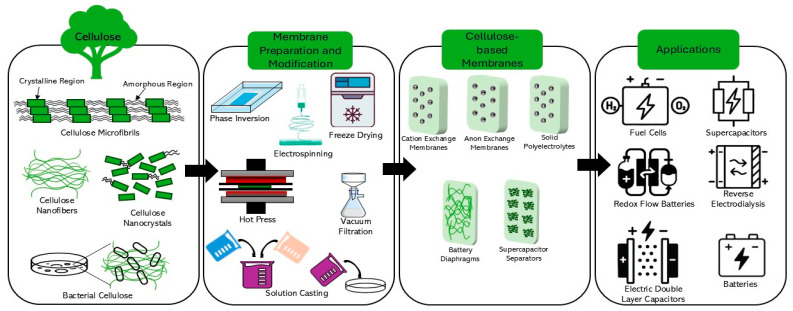
Schematic diagram of various cellulose forms, and preparation and modification methods of different cellulose-based membranes together with their applications in electrochemical devices.

The present article provides a comprehensive review of the various aspects of functionalized cellulose-based IEMs for electrochemical energy systems, including energy conversion and energy storage applications. The scope of the article offers an overview of various forms of cellulose-based membranes and strategies for the fabrication, together with the fundamentals of electrochemical energy conversion and storage systems, including the role of membranes and their performance in various polymer electrolyte membrane fuel cells (PEMFCs, DMFCs, MFCs, and AEMFCs), batteries such as LIBs, VRFBs, and supercapacitors, and RED. Finally, the challenges related to the performance and sustainability of the cellulose-based membranes, along with recommendations for future research directions, are also discussed. Figure 2 illustrates the schematic representation of cellulose forms, the synthesis of different cellulose-based membranes, and their applications in electrochemical devices.

## 2. Overview of Strategies for the Fabrication of Cellulose-Based Membranes

### 2.1. Fabrication Strategies for Producing Cellulose-Based Membranes

Various fabrication strategies have been used for producing cellulose-based membranes tailored to diverse applications. Common methods include dissolution–regeneration, functionalization followed by regeneration, and phase inversion. These techniques, which are widely used in the fabrication of membranes for separation and purification, have been comprehensively reviewed in the literature [29]. On the other hand, the fabrication of cellulose-based membranes for separation and electrochemical energy applications includes methods such as solution casting or phase inversion, which is used to produce membranes from regenerated cellulose or cellulose derivatives such as CMC, cellulose acetate (CA), hydroxypropyl methyl cellulose (HPMC), and methyl cellulose (MC) [29,31]. This method involves the use of toxic solvents such as N-methyl morpholine N-oxide (NMMO) or N, N-dimethylacetamide/lithium chloride (DMAc/LiCl) to dissolve the polymer and cast it into films, followed by solvent exchange. Electrospinning is another versatile method used to convert cellulosic polymers (e.g., CA) dissolved in solvents into cellulose nanofibers and nanofibrous sheets, which are subsequently functionalized. This technique produces highly porous scaffolds/membranes with excellent mechanical and ion transport properties, making them ideal as separators in LIBs [40] and supercapacitors [2,37]. Regeneration from native cellulose solutions with ionic liquids or alkali/urea aqueous systems can be used to produce dense, hydrophilic membranes that can be subsequently functionalized to produce PEMs and AEMs for fuel cells [41,42]. To enhance ion-exchange capabilities, in situ grafting techniques are commonly employed. These include radiation-induced graft copolymerization (RIGC) using high-energy radiation such as gamma rays or electron beam, and low-energy sources such as UV and plasma. Such approaches enable the direct introduction, or indirect incorporation through post-grafting reactions, of functional groups like sulfonic acid or quaternary ammonium onto the surface and the bulk of cellulose backbones [43,44]. This leads to potential polymer electrolytes serving as cost-effective and eco-friendly alternatives to PFSA membranes in applications such as fuel cells and water electrolyzers. Freeze-drying is another technique used to fabricate highly porous membranes. Cellulose nanocrystal (CNC) or nanofibril (CNF) suspensions can be employed to produce lightweight and high-surface-area structures that are suitable for VRFBs, catalytic supports, and supercapacitor electrodes [45]. Nanocellulose suspension can also occur under hot pressing or film casting to produce self-supporting membranes that can be subsequently modified with chemical groups to impart ion conductivity. Finally, cellulose or CNC can be used to form composite membranes by blending with synthetic polymers such as poly(vinylidene fluoride) (PVDF) [46], polyvinyl alcohol (PVA) [47], and poly(ethylene oxide) (PEO) or inorganic fillers, including silicone dioxide (SiO_2_) [48], titanium dioxide (TiO_2_), and graphene oxide (GO) [49]. The cellulose-based composite membranes provide tailored structures with excellent thermal and electrochemical properties suitable for a wide range of electrochemical systems. A comparison of the various methods used for the fabrication of cellulose-based ionic membranes is presented in Table 1. More details on various methods for the fabrication of cellulose-based membranes can be found elsewhere [27,29,31]. 

### 2.2. Modification Techniques for Fabrication of Cellulose-Based Membranes

Modification of cellulose to fabricate IEMs has been widely explored using several techniques, each providing unique benefits and challenges. They include (i) RIGC, (ii) direct chemical functionalization, (iii) polymer blending and crosslinking, and (iv) inorganic nanoparticle incorporation and surface functionalization of nanocellulose such as bacterial cellulose (BC), CNCs, CNFs, and cellulose nanowhiskers (CNWs) [50]. RIGC has been particularly effective, as high-energy irradiation generates radicals on cellulose backbones that initiate polymerization of ion-conducting monomers. Grafting can also be initiated using low-energy radiation, such as UV, in the presence of a photoinitiator or using plasma [51]. For example, grafting of vinylbenzyl chloride (VBC) onto cellulose, followed by quaternization, produced AEMs with high ion-exchange capacity and hydroxide conductivity [52], while styrene grafting followed by sulfonation yielded cation-exchange membranes (CEMs) with excellent proton conductivity and oxidative stability. Direct chemical functionalization of the hydroxyl group of cellulose provides another straightforward route for IEMs, with sulfonation introducing –SO_3_H groups for proton conduction [53,54] and quaternization introducing –NR_4_^+^ groups for alkaline-stable AEMs conductiong hydroxide ion [50,55]. Polymer blending and crosslinking [56] approaches, such as combining cellulose with Nafion [57] or chitosan-based quaternized polymers [58], have been shown to improve mechanical strength, dimensional stability, and ion-transport synergy, although phase compatibility remains a limitation. Inorganic nanoparticle incorporation has also been employed to enhance stability and ionic pathways; for instance, embedding sulfonated GO [59], TiO_2_, or SiO_2_ [48] into cellulose/derivatives matrices improved water retention, oxidative resistance, and proton conductivity, although excessive loading can compromise flexibility [60]. Surface functionalization of nanocellulose has emerged as a promising strategy due to the high surface area and tunable chemistry of nanoscale cellulose [61]. Quaternized CNCs yielded AEMs with superior alkaline resistance [62], while sulfonated CNFs provided flexible, high-ion exchange capacity (IEC) proton-conducting membranes with improved dimensional stability [63]. In summary, RIGC and chemical functionalization remain the most versatile for tailoring IEC and conductivity, blending, nanoparticle incorporation, and nanocellulose-based modifications offer complementary routes to optimize performance for fuel cells, redox flow batteries, and water treatment applications.

## 3. Overview of Cellulose-Based Membranes for Electrochemical Energy Systems

The use of cellulose for fabricating various types of membranes in electrochemical energy systems offers a sustainable and environmentally friendly alternative to conventional synthetic polymers. Such green membranes are employed as IEMs, PEMs, and separators for batteries and supercapacitors, thereby imparting both sustainability and cost effectiveness. Cellulose-based membranes incorporate different structure of cellulose as building blocks, including BC, cellulose derivatives (e.g., CA, cellulose nitrate, and ethyl cellulose), and extracted CNF and CNC [27]. The selection of the cellulose type, together with the fabrication method, controls the inherent properties of the membranes, including pore size, porosity, and ultimately its functional performance in electrochemical systems.

### 3.1. Various Cellulose Structures for the Fabrication of Cellulose-Based Membranes

Nanocellulose and its derivatives represent a promising class of sustainable nanomaterials with multifunctional properties and diverse applications. They possess exceptional mechanical strength, high surface area, and tunable surface chemistry. Nanocellulose exists in several forms: nanocrystalline cellulose (NCC), also known as CNCs, CNWs, CNFs, and BC as discussed earlier [64]. Fundamentally, these forms differ significantly in terms of morphology, production process, and degree of crystallinity [6]. BC offers exceptional purity, high crystallinity, and a robust nanofibrillar network that provides mechanical stability, high water uptake, and ion-transport capability, although it has dense structure and limited scalability restricting its widespread application [65]. NCC/CNCs in particular have a rigid rod-like shape and high surface area that make them suitable for enhancing stiffness and dimensional stability while providing abundant sites for functionalization [66]. They have found a wide range of applications, including environmental remediation (e.g., CO_2_ adsorption), water filtration, desalination, and water treatment at large [67,68,69,70,71,72], medical applications, such as wound dressings, tissue engineering scaffolds, drug delivery systems [73,74,75], and biosensors (due to their biocompatibility and ability to retain moisture), food packaging [76,77], flexible electronics [78,79], and electrochemical energy systems. However, if they are highly loaded, they increase the brittle nature and limit membrane processability. In contrast, CNFs form flexible, entangled fibrillar networks with abundant -OH, offering excellent mechanical reinforcement and tunable ionic conductivity, though their high aqueous viscosity and tendency to aggregate complicate membrane fabrication. CNWs with a greater aspect ratio and crystallinity than CNCs improve the dimensional stability and proton conductivity when functionalized [80]. The use of CNCs, CNFs, and CNWs in fabricating nanocomposite membranes involves their dispersion within a polymeric matrix or continuous phase, leading to different types of cellulose particle-reinforced polymer membranes and fiber-reinforced cellulose membranes, where they are combined with biopolymer or synthetic polymer substrates [81]. However, these applications face challenges of aggregation and poor dispersion during blending with other polymers. A comparison between the merits and demerits and imparted properties of various cellulose nanostructures for membrane applications is presented in Table 2. The polymer nanocomposites with various cellulose nanostructures as fillers or for reinforcement has properties that depend on the characteristics of the cellulosic nanofillers, polymeric matrices, and the interaction between them [30]. Polymer matrices can be natural polymers such as chitosan, alginate, and gelatine, or synthetic polymers such as PVA, PEO, and PVDF [82]. The synergy between cellulose and these polymer matrices permits designing membranes with multifunctional capabilities, balancing sustainability with high electrochemical performance.

### 3.2. Classes of Cellulose-Based Membranes

Various cellulosic forms can undergo chemical modifications to provide additional desired properties to enhance their potential applications and lead to functional materials such as IEMs. Cellulose-based IEMs can be classified based on their functional groups into four categories: AEMs, CEMs, amphiphilic membranes, and zwitterionic membranes. In cellulose-based AEMs, quaternary ammonium, imidazolium, or pyridinium groups are grafted to impart OH^−^ ion conductivity, mechanical stability, and alkaline resistance, enabling applications in AEMFCs, AEMWEs, and VRFBs [83]. Cellulose-based CEMs, modified by acidic moieties such as –SO_3_H and –COOH, dissociate to form a large number of protons, resulting in high proton conductivity associated with water retention and cation selectivity, making them suitable for PEMFCs and PEMWEs [3,84]. Cellulose-based amphiphilic membranes combine hydrophilic ionic groups (–OH, –SO_3_H, –COOH, and –N^+^R_3_) with hydrophobic moieties (alkyl or aromatic segments), promoting phase-separated morphologies that provide efficient ionic channels while improving dimensional stability and long-term durability under hydrated conditions, with applications in separation and oil/water purification advanced fuel cells, flow batteries, and selective ion separation [85,86]. Finally, Zwitterionic cellulose membranes are functionalized with both anionic (e.g., sulfonate, carboxylate) and cationic (e.g., quaternary ammonium) groups, resulting in balanced charge distribution, strong hydration layers, and antifouling properties, which are particularly useful in water purification, hemodialysis, and antifouling separation systems [87]. The present review, however, deals only with cellulose-based AEMs and CEMs, while other types of cellulose-based membranes primarily designed for separation and purification fall beyond the scope of this article.

## 4. Electrochemical Energy Conversion Systems

Electrochemical energy conversion systems involve the use of chemical reactions to convert chemical energy into electrical energy. The use of an electrochemical conversion system provides several advantages over conventional energy counterparts, including high efficiency, low emissions, modularity, flexibility, and scalability [88]. In principle, electrochemical conversion cells consist of an anode and a cathode separated by an IEMs, which regulate ion transport and fuel gas permeation. For instance, in the PEMFC, the membrane conducts ions from the anode to the cathode while preventing the mixing of reactant gases. Operating PEMFC above 100 °C provides a few advantages, including enhancement of the reaction kinetics, especially at the cathode, and a reduction in the complexity of the system, but such a system requires water-independent or anhydrous PEM [89]. If methanol is used as a fuel instead of hydrogen, the system is known as DMFC, which simplifies the design and makes it more suitable for portable devices. On the other hand, AEMFCs uses AEMs, which conducts hydroxide ions from the cathode to the anode and serves as a barrier to reactants. Replacing PEMs with AEMs opens the possibility of using non-precious metal catalysts, thus reducing system costs and aiding commercialization [90]. AEMFCs are superior to PEMFCs in several ways, including increased CO_2_ tolerance and less sensitivity to fuel contaminants. AEMFCs may be more durable and long-lasting than PEMFCs since AEMs are often more resistant to degradation from difficult working conditions [91].

AEMFCs usually use hydrogen as a fuel; however, the use of liquid fuels such as methanol has also been reported [92]. Another type of fuel cell using PEM is MFCs, which constitute a bio-electrochemical system that uses microorganisms to convert the chemical energy of the organic compounds, such as glucose, acetate, lactate, and wastewater, into electrical energy. The system operates at 20–35 °C, and the microbial oxidation of organic waste takes place at the anode, releasing electrons and protons. The electrons flow through an external circuit to the cathode, generating electricity, whereas protons are being transported via PEM. However, MFCs have much lower power output in the range of 0.01–0.3 mW cm^−2^ compared to 700–1000 mW cm^−2^ for PEMFCs [93,94]. Like PEMFCs, the PEM plays a similar key role in separating the anode and cathode compartments and enabling ion transport [95]. Unlike normal PEMs, MFC membranes must function in biologically active, low-temperature environments, allowing proton or cation transfer while maintaining anaerobic conditions at the anode. Thus, the MFC membrane must have additional properties such as biocompatibility with electroactive microbes, resistance to biofouling, and facilitating microbe-to-electrode electron transfer [96]. Like PEMFCs, Nafion^®^ membrane has been the most popular PEM that has been tested in dual chamber MFC on various occasions, and the performance was evaluated concerning the effect of pre-treatment and biofouling [97]. However, the membrane is still challenged by high cost and biofouling, and membranes with high performance and low cost are highly sought [98].

Energy conversion systems also include the use of electrolytic cells for hydrogen production from water using electricity that can be obtained from renewable sources. Such technology provides hydrogen, which is an energy carrier/fuel with a higher specific energy content than fossil fuels. Water electrolysis, therefore, plays an important role in the global energy transition and decarbonization of polluting industries. During water electrolysis, water is split into hydrogen and oxygen under electricity using an electrolyzer consisting of an anode, a cathode, separated by an electrolyte, and a power supply. The electrolyte is made of alkaline or acidic aqueous solution containing OH^−^ or H^+^ ions and can be substituted with AEM or PEM [99]. PEMWE typically operates under acidic conditions, utilizes highly conductive PEM that offers improved operational efficiency, and serves as a viable alternative to alkaline water electrolyzers. In contrast, AEMWEs operate in alkaline environments and allow the use of non-precious metal catalysts, potentially lowering system costs. Both PEMWE and AEMWE technologies are essential to advancing green hydrogen production and facilitating the integration of renewable energy into the power grid [100].

**Figure 3:**
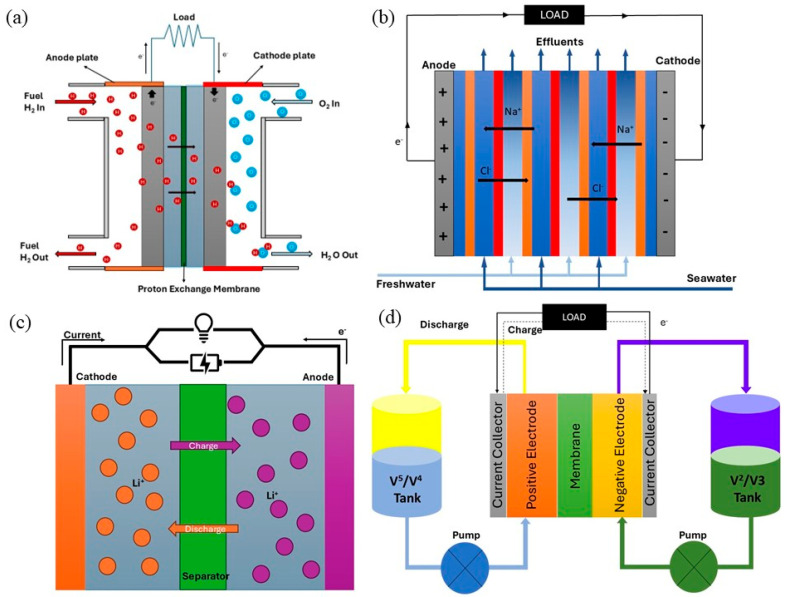
Schematic diagrams of (**a**) PEMFC, (**b**) RED, (**c**) LIB, and (**d**) VRFB.

RED is another promising electrochemical technology harnessing energy from salinity gradients resulting from the interaction of fresh water and salty water. This process uses IEMs (PEM and AEM) in separating the anode and cathode in electrochemical cells, in which the ion flows to generate potential differences that are converted into electricity [101]. RED systems rely heavily on the performance of the IEMs, where high permselectivity, low resistance, and minimal fouling are essential for maximizing power output. Cellulose-based and bio-derived membranes are gaining attention in RED due to their environmental compatibility, mechanical robustness, and potential for functional modification. On the other hand, pressure-retarded osmosis (PRO) is another mechanical process to harvest the salinity gradient by using water-selective membranes and turbines to produce electrical energy [102]. However, RED is more favorable for power generation because of its greater energy efficiency and reduced sensitivity to membrane fouling compared to PRO [103]. Figure 3 shows schematic diagrams for PEMFC, RED, LIB, and VRFB.

## 5. Energy Storage Systems

Energy storage systems (ESS) are devices that convert stored chemical energy into electrical energy by utilizing ion transfer in the membrane and electrons in electrodes. Despite having similar electrochemical components like fuel cells, batteries differ fundamentally in the way they function, where batteries are closed systems that store energy internally in chemical form and release it through reversible electrochemical reactions until the stored charge is depleted. Energy storage systems can be classified mainly into batteries and electrochemical capacitors. They are crucial for various applications, including powering portable electronics, electric vehicles, and grid-scale energy storage. IEMs are essential for facilitating ion transport, separating reactants, and preventing cross-over of active species in ESS [104]. Secondary LIBs are among the most advanced and widely adopted energy storage systems, extensively used in portable electronics, electric vehicles, and grid storage applications due to their high specific energy, excellent coulombic efficiency, and remarkable cycle life. These batteries are comprised of an anode, a cathode, an electrolyte, and a separator. The battery separator plays a critical role by preventing internal short circuits while allowing the free movement of lithium ions, thereby directly impacting the battery’s safety and power performance. Consequently, significant research efforts have focused on developing battery separators with enhanced mechanical strength, electrochemical stability, uniform pore structure, effective thermal shutdown behavior, and cost-effectiveness [105]. LIBs face safety concerns related to flammable electrolytes, thermal runaway, and dendrite formation at high currents. Research is ongoing to develop solid-state batteries using lithium ion-conducting polymer electrolytes that could mitigate these risks and improve safety, lifetime, and sustainability [106]. Of all such batteries, lithium-ion sulfate batteries (LISBs) are emerging as a next-generation high-energy-density [107].

VRFBs have received growing interest in energy storage systems due to their long cycle life, deep discharge capability, high energy efficiency, low cost, and scalability to the gigawatt (GW) level, which makes them most suitable for electric grids’ integration. A critical component of VRFBs is the IEMs, which serve as a barrier to block the crossover of vanadium ions while enabling proton transport to maintain electrical conductivity [108]. An ideal IEM should exhibit low vanadium ion permeability to reduce self-discharge, high ionic conductivity, strong chemical stability, and be cost-effective. However, currently available commercial membranes fall short of meeting all these criteria, and there is an ongoing demand for IEMs with high conductivity, stability, and vanadium barrier properties [109].

Supercapacitors offer another class of energy storage devices that bridge the gap between conventional capacitors and batteries. They are capable of rapid charge/discharge cycles, possess high power densities, and have excellent cycle stability. Supercapacitors are typically categorized into electric double-layer capacitors (EDLCs) and pseudo-capacitors, depending on their charge storage mechanism. Despite their lower energy density compared to batteries, the development of innovative electrode materials and high-performance separators/PEMs, including cellulose-derived components, can contribute to significant performance improvements and broaden their application in hybrid energy systems and wearable electronics [110]. Compared to conventional capacitors and batteries, supercapacitors are energy storage devices that can store more energy, deliver power faster, and surpass charge/discharge capabilities, which have attracted growing interest in this system [111]. Supercapacitors consist of electrodes, a separator/membrane, and an electrolyte, where the supercapacitor’s energy storage mechanism mostly consists of the physical adsorption after charging and the electrodes adsorb cations/anions to form the double-layer electric [112]. Out of the three components, the separator is crucial to prevent internal short circuit between anode and cathode, as well as ensuring free ion transport throughout the interlinked electrolyte-soaked structure [113].

## 6. Cellulose-Based Membranes for Fuel Cells

### 6.1. Proton Exchange Membranes for Proton Exchange Membrane Fuel Cells

The PEM is a critical component in PEMFC for conducting protons from the anode to the cathode, separating the reactants, and preventing electron transport. Current PEMFCs typically use PFSA membranes like Nafion^®^ (DuPont, Wilmington, DE, USA) and alternatives such as Aciplex (Asahi Kasei, Kawasaki, Japan), Flemion (Asahi Glass, Kameido, Koutou-ku, Tokyo, Japan), Gore-Select^®^ (W. L. Gore & Associates, Newark, DE, USA), Hyflon^®^ Ion (Solvay, Bollate, Italy), and Fumapem^®^ (Fumatech BWT GmbH, Bietigheim-Bissingen, Germany) [114]. Despite the advantages of these membranes, their cost is still considered high, and their production is energy-intensive with a significant environmental footprint. The incorporation of perfluorinated components complicates disposal and raises environmental sustainability concerns. Thus, new alternative membranes continue to be in demand. The new membrane should attain combinational properties, such as high proton conductivity, low fuel permeability, low electronic conductivity, good chemical and thermal stability, mechanical strength, and affordability. As a result, significant research has focused on developing an improved variety of PEMs [115]. One appealing method that reduces the environmental burden and meets sustainability goals that has been widely adopted is to use nanocellulose structures in the forms of CNCs, CNFs, and nanocrystalline BC with their outstanding mechanical characteristics and versatility to develop cellulose-based composite membranes with heterogeneous structures for fuel cells. This included crucial treatments involving not only physical treatment (impregnation) but also chemical modifications of cellulosic materials or their combination with conductive materials to impart ionic conductivity and gas barrier properties required for PEMs [115].

The use of cellulose for the development of alternative biobased PEM has been recognized for more than a decade. An early example involving the fabrication of cross-linked PEM was by blending cellulose and sulfosuccinic acid (SA), followed by thermal esterification of –OH of the cellulose and –COOH of SA, as reported by Seo et al. [116]. The content of SA affected the electrochemical properties of the membrane (Cell/SA), which displayed a maximum proton conductivity of 23 mS cm^−1^ at 30.0 wt.% of SA concentration. Similar membranes were recently reported based on cellulose extracted from an agricultural byproduct (rice husk) and sulfonated using deep eutectic solvents as the modification. The membranes demonstrated high proton conductivity in the range 1.79–2.98 mS cm^−1^ [117]. No further details were reported on such membranes. Another biobased PEM comprising 2,2,6,6-tetramethylpiperidine-1-oxyl (TEMPO)-oxidized and sulfonated CNF displayed interesting properties, including water uptake of 102% and proton conductivity of 1.75 mS cm^−1^, which are comparable with counterparts reported in literature [118].

BC is one form of cellulose that has been frequently used as a substrate for the development of bio-based IEMs with appealing properties [119]. Several studies reported groundbreaking results using modified cellulose for the fabrication of PEMs for potential fuel cell applications. The graft polymerization method was used to modify BC with proton-conducting groups. In a study reported by Lin et al. [120], a PEM was prepared via graft polymerization of 2-acrylamido-2-methyl-1-propanesulfonic acid (AMPS) on nanocrystalline BC with UV irradiation. The BC/AMPS membrane, with a 39.5% degree of AMPS grafting, exhibited high-water uptake of 264% and an IEC of 1.79 mmol g^−1^ together with a 29 mS cm^−1^ proton conductivity. The membrane displayed a maximum power density of 97 mW cm^−2^ at 50 °C. Methyl methacrylate (MMA) was also grafted on CNF, yielding (CNF-*g*-PMMA) and used to modify phenol formaldehyde resin (PF), forming a membrane with improved structure, enhanced conductivity (542 mS cm^−1^), and improved mechanical properties [121].

**Figure 4:**
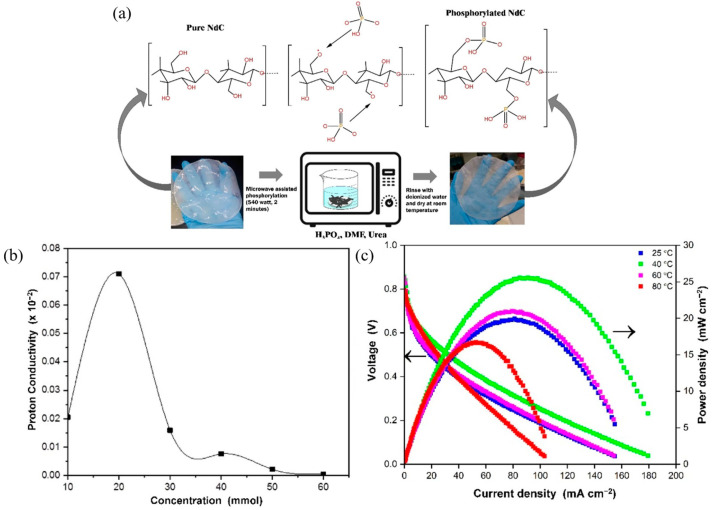
BC-based membranes: (**a**) procedure of fabrication from Nata de Cassava, (**b**) proton conductivity of the membrane at various concentrations, and (**c**) performance of the membrane in a single cell set-up at different temperatures. Reprinted with permission from Kartika Sari et al. [126].

Chemical modification was also used to prepare a membrane comprising carboxymethylated bacterial cellulose (CMBC) combined with polyaniline (PANI) and 4-polystyrene sulfonic acid (PSSA) to yield CMBC/PANI [122] and BC/PSSA membranes [123], respectively. CMBC/PANI membrane exhibited relatively low ionic conductivity (25.2 mS cm^−1^), which might restrict its suitability for high-power-density fuel cell applications. In contrast, BC/PSSA was an ultra-thin (0.1 μm) membrane, suggesting potential advantages in applications where minimal thickness is crucial. The properties of the BC/PSSA nanocomposite membrane were improved and delivered one of the highest power densities among bio-based PEM in fuel cells (40 mW cm^−2^ at 125 mA cm^−2^). A similar BC/PSSA nanocomposite membrane exhibited high thermal stability (up to 155 °C), mechanical integrity coupled with ion exchange capacity of 1.85 mmol g^−1^, and proton conductivity of 1.73 mS cm^−1^. The membrane yielded a maximum power density of 2.42 mW cm^−2^ when it was tested in a single-chamber lab-scale MFC using a *Shewanella frigidimarina* pure culture [123]. An enhanced proton conductivity of 185 mS cm^−1^ (at 40 °C, 98% RH, in plane) was reported for a similar BC/PSSA membrane [124]. Earlier, the potential of BC/Nafion nanocomposite membrane with a 100 μm thickness and a proton conductivity of 140 S cm^−1^ (at 94 °C and 98% RH) was evaluated in PEMFC [125]. Figure 4 represents steps of fabrication and testing of a membrane made of BC synthesized from *Cassava* and microwave-assisted phosphorylation process (Figure 4a) produced 0.4154 μm thickness. The membrane with 20mmol phosphoric acid concentration produced the highest ion conductivity 71.0 mS cm^−1^ at room temperature (Figure 4b). The membrane performance in a single-cell setup resulted in a high power density of 25 mW cm^−2^ at 40 °C which can be seen from Figure 4c that shows the polarization curve (left arrow) and power density (right arrow) profile at various operating temperature [126].

A series of PEMs based on chemically modified BC with different functionalities was reported previously [127,128,129].The first PEM based on BC modified with crosslinked poly (methacryloyloxyethyl phosphate) (PMOEP) was fabricated [103]. The membrane was a nanocomposite made of BC modified by poly(bis [2-(methacryloyloxy)ethyl] phosphate) (P(bisMEP)/BC) fabricated by the in situ free radical polymerization of bisMEP inside a 3-D network structure of BC, which demonstrated an interesting combination of thermal stability (up to 200 °C), mechanical integrity, and IEC (up to 3.0 mmol g^−1^) and proton conductivity of ca. 30 mS cm^−1^ [128]. The second membrane was made with a thickness of 42 μm and a high ionic conductivity of 100 mS cm^−1^. However, the BC/PMOEP membrane attained an excessive water uptake (206%), which could potentially enhance proton transport but compromised its mechanical strength and disqualified it for fuel cell applications [104]. The third membrane was a fully bio-based membrane in which BC was combined with an algae sulphated polysaccharide known as fucoidan (Fuc), forming a fully biopolymer-based PEM (BC/Fuc) that exhibited reasonable thermal, mechanical, and proton conductivity properties [129]. The maximum proton conductivity was 1.6 mS cm^−1^ at 94 °C and 98% RH.

In another membrane, N-butylguanidinium tetrafluoroborate (BG-BF_4_), an aprotic ionic liquid, was also used to impregnate the BC network to fabricate a PEM with different combinations. Overall, the 95 wt% BG-BF_4_ containing membrane exhibited a reasonable ionic conductivity of 0.52 mS cm^−1^ at 180 °C but a reduced mechanical strength (6 MPa). Further modification was carried out by incorporating PANI into the BC matrix via oxidative polymerization, which improved the membrane properties and provided a water-free BC-based composite membrane for fuel cells [129,130]. Another biobased PEM was fabricated by the combination of lignosulfonates (LS) with nanocrystalline BC to form (Ls/BC) membrane that exhibited ionic conductivity of 23 mS cm^−1^ at 94 °C and RH of 98% [131]. A revision of the performance of several BC-based PEMs for fuel cell application can be found elsewhere [124].

The potential of sulfonated kraft lignin (LSx) as a fully biobased membrane for the application as a proton conductor was reported by Farzin et al. [132]. The LS 1.6/Na_2_SO_3_ attained an ionic conductivity about an order of magnitude higher than Nafion in submicron-thick films, suggesting the great potential of lignin-based PEM. Nano BC and LS were blended via the diffusion of an aqueous solution of the lignin derivative and tannic acid as a crosslinker into the wet BC nanofibrous 3-D structure to produce a fully biobased PEM. The BC/LS membrane attained good mechanical stability and displayed a maximum of 23 mS cm^−1^ at 94 °C and RH of 98%, showing its potential for PEMFC. More details on studies on various types of cellulose-containing membranes based on BC can be found elsewhere [18].

Microcrystalline cellulose (MCC) and NCC/whiskers and micro- and nanofibers with their unique properties have also been used as fillers, matrix, or reinforcement component for the development of the composite IEMs with enhanced properties [133]. Bayer et al. [134] was first to report the fabrication of carboxylated CNF and CNC PEMs for fuel cells. The membrane demonstrated a maximum ionic conductivity of 0.01 mS cm^−1^ at 100% RH and 30 °C. The conductivity was further increased to 0.05 mS cm^−1^ at 100 °C, and this was coupled with good H_2_ barrier properties. However, the membranes were still water-dependent and did not support high-temperature PEMFC operation despite their successful test up to 80 °C. A similar carboxylated CNF membrane was reported by Guccini et al. [135]. The high affinity of CNF for water and the developed well-defined and homogenous membrane structure imparted a proton conductivity exceeding 1 mS cm^−1^ at 30 °C between 65 and 95% RH.

**Figure 5:**
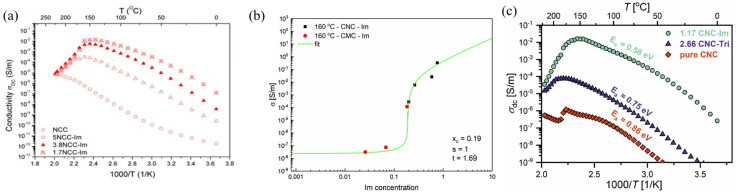
Properties of NC-based membranes: (**a**) DC conductivity vs. inverse temperature for NCC-Im membrane [137], (**b**) electrical conductivity of CNC-IM as a function of conductive imidazole content compared with earlier studies [138], and (**c**) conductivity of CNC, 2.66−CNC-T Tri, compared to 1.17−CNC-IM composite membranes [139]. Reprinted with permission from Tritt-Goc et al. [138].

Smolarkiewicz et al. [136] reported the preparation of a composite membrane by modifying MCC or cellulose microfibers functionalized with N-containing molecules such as imidazole (Im), which was denoted as MCC-Im. The use of Im, having a high melting point, as an alternative agent for membrane functionalization allowed water-independent conductivity (>100 °C) that reached a maximum value of 0.000021 mS cm^−1^ at 150 °C, which is below the requirements for fuel cell application. Further improvements were made to this type of membrane by controlling the chemical modification to 1.0 Im per 1.7 glucose unit (1.7NCC-Im) as reported by Tritt-Goc et al. [137]. The obtained composite membrane showed conductivity as high as 0.27 mS cm^−1^ at 140 °C under anhydrous conditions, making it a promising candidate for various fuel cell applications (Figure 5a). The conductivity was further enhanced for 1.3 CNC-Im composite membrane with the highest ion conductivity (4 mS cm^−1^) despite its comparatively thicker thickness (0.234 mm) (Figure 5b) [138]. However, this membrane had a durability drawback under high temperature, and thus 1,2,3-triazole (Tri) was introduced as a replacement for Im [139]. The obtained 2.66 CNC-Tri composite membrane, having the ratio of concentrations molar glucose unit/triazole of 2.66, demonstrated maximum proton conductivity of 0.01 mS cm^−1^ at 175 °C under dry conditions, which is inferior to the 1.17 CNC-Im counterpart (Figure 5c). However, the 2.66 CNC-Tri composite membrane has far better thermal properties in terms of stability of the matrix and durability of the Tri molecule and demonstrated lifetime (5000 h at 120 °C and 200 h at 150 °C) meeting the requirements of PEMFC for mobility applications. Another tri-based sulfonated CNC composite was also reported by Etuk et al. [140]. A high proton conductivity of 1.3 S cm^−1^ at 120 °C was observed, but it drastically dropped above 100 °C, and it was not clear if measurements were performed under wet or anhydrous conditions.

Carboxylated membranes based on NCC, CNF, and MCC are another type of PEM that have been investigated on several occasions. Jankowska et al. [141] reported a comparison between the physicochemical properties of carboxylated membranes based on NCC, CNF, and MCC. After evaluating membranes’ properties, they obtained a maximum proton conductivity of 1.0 mS cm^−1^ at 90 °C without controlling RH conditions. In another study to improve conductivity, the carboxylate-modified NCC (COOH−10/NCC-5) membrane was fabricated with a thickness of 70 µm and exhibited relatively higher ionic conductivity (2.18 S cm^−1^). The nanoscale structure in this membrane contributed to improved performance, although its mechanical durability requires further evaluation.

To enhance the desired properties for fuel cell applications, cellulose has also been blended with several synthetic polymers, such as poly(aryl ether ketone) (PAEK) [56], PES [142], poly(ether ether ketone) (PEEK) [143], poly(ether ether ketone ketone) (PEEKK) [144], PBI [145], PSF [146], PVDF [147], PANI [130], and Nafion [57]. For instance, BC was used to fabricate a composite membrane by incorporating it into the Nafion matrix by Jiang et al. [148]. The membrane attained a maximum power density of 100 mW cm^−2^ in PEMFC compared to 60 mW cm^−2^ for the pristine Nafion membrane. 

Composite membranes of sulfonated poly(arylene ether ketone) (SPAEK) copolymers reinforced with NCC (5%) and functionalized with 10% and 30% –COOH were also reported [149]. The SPAEK-COOH10/NCC5 (5% NCC) membrane exhibited a higher proton conductivity of 272 mS cm^−1^ than that of SPAEK-COOH-30/NCC, which reached 0.239 S cm^−1^ at 100 °C, but the latter showed better mechanical properties. It was found that the NCC-enhanced composite membranes were better in proton conductivity and mechanical properties compared with the pristine COOH-10 membrane [56].

CNFs were also used to fabricate membranes with improved proton conductivity via their incorporation with phosphoric acid and combining with sulfonated poly (ether sulfone) (SPES) matrix by Cai et al. [142]. Turning to physically modified composite membranes (P-CNF/SPES) with moderate thickness (85–120 μm) conceived an ionic conductivity of about 154 mS cm^−1^ at 80 °C and 100%RH, coupled with notable water uptake of 45%. Similar CNF/SPES with 5% CNF demonstrated proton conductivity of 0.13 S cm^−1^ at 80 °C and 100% RH [150].

Fluorine-containing sulfonated polybenzimidazole (s-PBI)/cellulose/silica (s-PBI/Cell/SiO_2_) is a notable biocomposite membrane, with a thickness range of 45–55 µm and strong ionic conductivity of 9.11 mS cm^−1^. Because of its low thickness and excellent conductivity, this membrane has a strong potential for PEMFC [151].

Composite membranes based on CNCs, CNFs, and PBIs were further compared to uncover the underlying differences in their performance for PEMFC [145]. The NCC-Im membrane achieved a higher conductivity of 0.326 S m^−1^ at 150 °C than that of its counterpart (NCF-Im), which was 21.0 mS cm^−1^ at 140 °C. Zhao et al. [144] reported an investigation of a composite membrane based on sulfonated poly (ether ether ketone ketone) (SPEEKK) and chemically modified cellulose. The authors changed MCC to sulfonated NCC, which was further modified with amino (Am) groups using 3-trimethoxysilyl propyl ethylenediamine. The modified Am-sNCC were blended into S-PEEKK, forming a composite membrane that exhibited higher proton conductivity, reaching a maximum value of 0.210 S cm^−1^ at 100 °C at 5 wt% in Ph-PEEKK/Am3-sNCC. Another nano-composite membrane was composed of SPEEK crosslinked and reinforced with ethylene glycol (EG) and CNCs by Bano et al. [143]. The crosslinked CNC/SPEEK membrane with 4% CNC loading and 70 µm thick has a proton conductivity of 0.186 S cm^−1^ at 95 °C and 95% RH. The membrane material established a structural combination capable of balancing proton transport and mechanical integrity. An outstanding improvement in SPEEK-based membrane blended with sulfo ethyl cellulose (SEC) of 10 wt% showed 109.2 mS cm^−1^ proton conductivity and 30% water uptake, which are highly promising for PEMFC [152].

Reduced graphene oxide/polyvinylidene fluoride (CA-rGO-PVDF), a superior cost-effective PEM integrating PVDF, CA, and rGO for a microbial fuel cell, was reported by Sharma et al. [147]. The thickness of CA-rGO-PVDF was 120 μm, and its ionic conductivity reached 0.4 S cm^−1^. Other cellulose-based membranes, such as S-CNFs and CA-GO, displayed distinct properties. For example, S-CNFs, with a thickness of 30 μm, attained a good balance of thickness and ionic conductivity (2 mS cm^−1^), making them useful for MFC. CA-GO, on the other hand, with a thickness of 25 μm, demonstrated an outstanding ionic conductivity of 15.5 mS cm^−1^ coupled with a high-power density of 519 W m^−1^. This membrane provided an improvement in the overall performance by facilitating mass transfer in fuel cells. Another nanocomposite membrane using sulfonated GO (SGO) embedded into the NCC/PVA matrix was reported by Muhmed et al. [153]. The adopted combination, in which increasing the SGO loading improved the properties, led to the highest proton conductivity of 11.0 mS cm^−1^ for NCC/PVA-SGO-1.0 at 80 °C under 100% RH. The membrane exhibited a power density of 31.4 mW cm^−2^ and a current density of 60.2 mA cm^−2^ when tested in PEMFC. The performance of this membrane was superior to the NCC-50/PVA-50 membrane reported by the same authors, which displayed proton conductivity of 30 mS cm^−1^ [154].

PEM based on MCC from cellulose filter paper (cheap substrate) reinforced with resorcinol bis (diphenyl phosphate) (RDP) achieved a maximum power output of 4.9 mW cm^−2^ at 80 °C. When cellulose filter paper was treated first with a mixture of phosphoric acid and citric acid, and RDP, it led to the highest power enhancement of 226% in power output, reaching 16 mW cm^−2^ (in air) and 34.3 mW cm^−2^ in O_2_ [155]. Furthermore, the MCC/RDP/PO_3_–H membrane was stable under a constant current of 60 mA for at least 100 h with only an 8% loss. Table 3 presents a summary of previous studies on cellulose-based membranes with various fabrications and associated characteristics for PEMFCs.

Based on discussion on previous studies, it can be observed that functionalized cellulose-based PEMs for PEMFCs seem to be cost-effective and sustainable. Functionalization strategies such as carboxylation, sulfonation, or phosphorylation enhance proton conductivity but often come with high water uptake and swelling, compromising mechanical and dimensional stability under humid PEMFC operating conditions. Crosslinking is applied to decrease swelling and improve structural strength, but can restrict polymer chains’ mobility if excessively used, decreasing proton transport efficiency. The addition of reinforcement nanomaterials like CNCs, GO, Mxene, or inorganic oxides (SiO_2_) enhances mechanical strength and resistance to oxidation, but adds defects or non-uniformity if the dispersion is poor or compatibility with the cellulose matrix is poor. Moreover, such functionalized membranes are usually challenged by the inhomogeneous distribution of functional groups, making the conductivity within the membrane non-uniform. This means the membranes suffer from relatively low inherent proton conductivity due to a lack of ordered proton transport pathways. Moreover, variations in the cellulose sources used to prepare different forms and their processing methods pose a challenge to reproducibility and scalability. In addition, most of the functionalization and reinforcement techniques depend on complex, non-green chemistry processes that might compromise large-scale and low-cost fabrication. Finally, however, most of the studied membranes are based on short-term performance, and very little are made for long-term stability under dynamic conditions. Therefore, more efforts should be made to optimize the fabrication and functionalization parameters to ensure a balance between high ion transport and excellent mechanical integrity and chemical stability is maintained in cellulose-based PEMs for PEMFC.

### 6.2. Proton Exchange Membranes for DMFC

PEMs for applications in DMFCs face several persistent challenges hampering the commercialization of DMFCs, including sluggish methanol electrochemical oxidation, methanol crossover, cathode flooding, and massive noble metal use [164]. The most notable of these issues is methanol crossover, which occurs when methanol disperses through the membrane to the cathode, inhibiting performance at the cathode due to mixed potentials associated with methanol oxidation and carbon dioxide at the anode [164]. Nafion membranes were reported to have serious weaknesses, such as high methanol permeability and a limited operating temperature (<100 °C), in addition to high cost. Therefore, extensive efforts have been devoted to developing alternative materials for composite membranes, including cellulose-based PEMs. The membranes involved the use of different cellulosic forms, including MCC, NCC, and CNF or their modified forms, as a filler or reinforcement of various functionalized polymer matrices to provide modification of Nafion [164]. The most notable of these issues is methanol crossover, which occurs when methanol diffuses through the membrane to the cathode, inhibiting performance at the cathode due to mixed potentials associated with methanol oxidation and carbon dioxide at the anode [164]. Nafion membranes were reported to have serious weaknesses, such as high methanol permeability and a limited operating temperature (<100 °C), in addition to high cost. Therefore, extensive efforts have been devoted to developing alternative composite membranes, including cellulose-based PEMs. These membranes involved the use of different cellulosic forms, including BC, MCC, NCC, and CNF or their modified forms as fillers or for reinforcement of various functionalized polymer matrices, including modifications of Nafion membranes. 

According to Lin et al. [120], the modification of commercial PEMs (such as Nafion membranes) and commercial composite counterparts with PFSA functionality provides a path for improvements in performance, along with cost reductions. Composite Nafion membranes were formed by loading CNC in the form of CNWs into the structure of the Nafion matrix without thermal treatment. This led to the enhancement of the proton conductivity and improved the methanol barrier properties of the membrane [165]. Moreover, the membrane displayed a maximum power density of 91 mW cm^−2^ in a direct methanol/air fuel cell using a 5 M methanol solution at 70 °C compared to 47 mW cm^−2^ for the pristine Nafion membrane.

A bio-inspired PEM for potential use in DMFC was prepared and tested by Lin et al. [120]. These authors developed a grafted AMPS-*g*-BC membrane prepared by UV graft copolymerization of AMPS. The membrane demonstrated a high ionic conductivity of 29.0 mS cm^−1^ that was coupled with a 42% reduction in methanol permeability compared to Nafion 115. This is far greater than most physically modified cellulose-based membranes. Another membrane that incorporated amino acid (AA)-functionalized CNWs into a sulfonated polysulfone (SPSF) matrix was studied. The membrane with 10 wt% L-Serine-functionalized CNWs (CNWs/SPFS/AA) could achieve a maximum proton conductivity of 0.234 mS cm^−1^ at 80 °C [146]. The membrane also maintained a good combination of water uptake and reduced methanol barrier properties. These membranes were improved by Zhao et al. [144] by incorporating proton-conducting fluorenylmethoxycarbonyl (Fmoc) group amino acid (FAA) clusters onto CNF, followed by embedding in a sulfonated polyethersulfone (SPES) substrate. This step improved the water uptake and swelling of the membranes (CNF/SPES/AA) to manageable values restricted to 60 wt% and 30%, respectively. The CNF/SPES/FAA exhibited the highest ionic conductivity of 0.213 mS cm^−1^ at 80 °C under 100% RH at 10% serine, which increased to 0.264 mS cm^−1^ when serine content was increased to 20 wt%. These membranes exerted an excellent performance in DMFC with a maximum power density of 87.22 mW cm^−2^ at 60 °C under 100% RH using 2M methanol as a fuel. 

Membranes fabricated from PVA, chitosan (CS), and CNCs with improved thermo-methanol barrier properties for DMFC displayed low methanol permeability (3.12 × 10^−8^ cm^2^ s^−1^) compared to the pristine PVA membrane, with a methanol permeability of 4.19 × 10^−7^ cm^2^ s^−1^ [166]. However, protonation of the PVA-CS-CNC membrane with acids resulted in low proton conductivity in the range of 10^−4^ S cm^−1^. CNF functionalized with amino acids and metal organic frameworks (MOFs), and embedded into sulfonated PSF, was reported by Wang et al. [167] as a proton-conducting membrane CNF-UiO-66-NH_2_/SPF. The composite membrane with 5 wt% (nominal) UiO-66-NH_2_ displayed a proton conductivity of 0.196 mS cm^−1^ at 80 °C and 100% RH. This was coupled with controlled swelling of 17.3% and low methanol permeability coefficient of 5.5 × 10^−7^ cm^2^ s^−1^. 

Recently, NCC has been mixed with PVA, forming a biopolymer nanocomposite membrane, NCC/PVA, through solution casting under varying proportions. The NCC-50/PVA-50 membrane with a composition of 50% NCC and 50% PVA showed high water adsorption and fuel methanol crossover reduction. This membrane proved to have the best performance among all the composite membranes with a maximum proton conductivity of 3.0 mS cm^−1^, power density of 10.3 mW cm^−2^, and current density of 22.2 mA cm^−2^. Fuel cell tests revealed that the open-circuit voltage of the fuel cell with the membrane was stable at 0.98 V, indicating a good barrier property against methanol crossover [154]. On the other hand, combining MCC with 2,3-dialdehyde cellulose (DAC) with the SPEEK matrix followed by solution casting yielded a composite membrane with proton conductivity in the range of 0.1 to 0.127 mS cm^−1^ in a temperature range of 35–110 °C coupled with improved barrier properties against methanol [168].

A composite membrane made of MCC modified with phosphotungstic acid (PTA) and imidazole (Im) was prepared by the phase inversion method [169]. The obtained membrane displayed fair conductivity of 0.214 mS cm^−1^ at room temperature, coupled with improved methanol barrier properties. It was concluded that the inclusion of Im and PTA in the NC membranes enhanced IEC, boosting higher proton conductivity. In another study, Priyangga et al. reported ternary-component membranes composed of NC, Im, and mesoporous m-PTA [170]. The NC-Im-m-PTA-5 membrane showed an interesting combination of water uptake (50.68%), IEC (1.885 mmol g^−1^), proton conductivity (31.88 mS cm^−1^), and selectivity (1.83 × 10^4^ S cm^−3^). Furthermore, the membrane demonstrated reduced methanol permeability (1.74 × 10^6^ cm^2^ s^−1^) and methanol uptake (3.19%).

Sriruangrungkamol and Chonkaew [171] reported CNF-based membranes loaded with SSA at different concentrations. The membrane donated as NC-10SSA possessed an IEC of 0.069 mmol g^−1^ coupled with an enhanced proton conductivity of 3.2 mS cm^−1^ and a decreased methanol permeability of 1.95 × 10^−6^ cm^2^ s^−1^. It was revealed that SSA with CNF promotes crosslinking and the formation of hydrophilic ionic domains, which is the primary reason for the high performance of CNF-based membranes. Another SSA cellulose-based nanocomposite membrane with interesting properties was also reported recently by Sriruangrungkamol et al. [172]. The membrane was prepared via the modification of CNFs with SSA before loading into a sulfonated poly (ether imide) (SPEI) solution and subsequent casting to form the sulfonated CNF/SPEI composite membranes (*x*SCNF/SPEI). The 0.8SCNF/SPEI membrane exhibited an optimum combination of proton conductivity of 16.68 mS cm^−1^ and IE1 1.01 meq g^−1^ coupled with methanol permeability of 8.22  ×  10^−8^ cm^2^ s^−1^, which is two orders of magnitude lower than Nafion117 membrane. The sulfonated CNF/SPEI composite membrane exhibited a power density of 4.32 mW cm^−2^ when tested in DMFC at 80 °C. Another version of membrane using cellulose-based carbon nanodots (CNDs) for the fabrication of SPSF by Permana et al. [173]. SPSF/CND-1 containing 1 wt% of CND demonstrated the highest proton conductivity of 35.5 mS cm^−1^ and a low methanol permeability of 3.50 × 10^−6^ cm^2^ s^−1^ coupled with high selectivity. A comprehensive review of earlier studies on cellulose-based membranes for DMFC was published earlier [174]. A summary of previous studies on cellulose-based PEM membranes for DMFCs is shown in Table 4.

Based on previous studies on cellulose-based membranes, it can be observed that chemical modifications tend to yield higher ionic conductivity and lower methanol permeation compared to physical modifications, although the latter also enhanced the methanol barrier properties and selectivity compared to Nafion membranes. However, these membranes are still facing critical challenges in DMFC applications. One of the main challenges is the proton conductivity-selectivity trade-off, where the tendency to block methanol also limits the mobility of water and thus undermines the proton transport path, giving lower conductivity compared with the Nafion membrane. The functional groups (SSA, SA, PTA, etc.) introduced to the membranes to increase proton conductivity are subject to leaching or degradation in the oxidative environment of the DMFC, and this is likely to slow its performance. Some cellulose-based membranes that undergo excessive filler incorporation or reinforcement may experience a decrease in the mechanical integrity over the long term. Moreover, no stability evaluation for the membranes under dynamic conditions was reported in most of the published studies.

### 6.3. Anion Exchange Membranes for AEMFC

AEM plays a crucial role in AEMFC, where it serves as a barrier to prevent mixing of fuel and oxidant, while allowing the transport of OH^−^ ions from the cathode to the anode. However, AEMFCs must yet overcome several challenges for large-scale applications, such as the need for more effective anode and cathode catalysts and the development of stable materials in alkaline medium and cheap AEMs [178]. Since the performance of AEMFC mainly depends on the properties of AEM, various strategies have been adopted to prepare AEMs with enhanced properties such as high alkaline stability, excellent anion (OH^−^) conductivity, high mechanical and thermal stability, and cost-effective membranes over the past decade [179]. Alternative AEMFCs have produced encouraging results, with some showing high power densities and efficiency on par with PEMFCs. For instance, AEMFC with the polynorbornene-based AEM demonstrated the highest power density (3.5 W cm^−2^), which was coupled with a maximum durability of 2000 h of operation at the single-cell level [180]. Despite the progress that is being made in the development of AEMs, there are still significant efforts to be made to enhance performance and address durability challenges that are hindering the deployment of large-scale applications [3].

Various forms of cellulose were used to prepare AEMs with cationic groups such as quaternary ammonium (−NR_3_^+^). Zeng et al. [181] reported the fabrication of composite AEM by BC-loaded TiO_2_ functionalized with quaternary ammonium groups to prepare 3-chloro-2-hydroxypropyl trimethyl ammonium chloride (CHPTAC) [181]. The mechanical properties of the composite films were improved by the addition of PVA, and the obtained BC/TiO_2_/CHPTAC-OH/PVA displayed a modest ionic conductivity of 93.0 mS cm^−1^ at 80 °C. In another study, an alkanized BC-poly(diallyldimethylammonium) chloride composite was also prepared based on BC, and the obtained BC-PDDA-OH- membrane was proven to have mechanical integrity and high -OH^−^ conductivity, reaching a value of 51 mS cm^−1^, supporting their potential application AEMFC [182].

In another interesting study, in situ free radical polymerization was used to prepare nanocomposite membranes of poly(methacroylcholine chloride) (PMACC) and BC [183]. The resulting BC/PMACC membrane demonstrated a remarkably high ionic conductivity of 10 mS cm^−1^. The membrane appeared to be quite successful in boosting ion transport. BC was also used as a porous substrate with layered double hydroxide (LDH) to develop AEM with a balance between ionic conductivity, mechanical properties, and alkaline stability, as reported by Nie et al. [184]. Membrane fabrication involved pore-filling of an ionomer containing both quaternary ammonium and a hydroxyde group, along with sol–gel crosslinking using organosiloxane. The obtained membrane exhibited a tensile strength of 47.48 MPa that was 88.3% higher than that of the pure ionomeric membrane and showed an ionic conductivity of 70.7 mS cm^−1^ (at 80 °C). The BC/LDH demonstrated excellent alkaline stability, as indicated by the 85.5% residual conductivity ratio after hot KOH treatment for 580 h. The membrane exhibited a peak power density of 13.6 mW/cm when tested in an alkaline direct methanol fuel cell.

Another approach for preparing AEM was adopted by Yu et al. [62] who introduced multilayer coating to a BC porous substrate with TiO_2_, with subsequent functionalization by quaternization, which was then in situ filled and polymerized with benzyl vinyl trimethyl ammonium chloride (VBTAC), which is an ionic liquid, to obtain AEM with simultaneously enhanced ionic conductivity and mechanical properties via a simple and environmentally friendly route. The BC/TiO_2_/VBTAC (PIL/Q TiO_2_/BC) membrane achieved an excellent combination of properties, including ionic conductivity of 100.5 mS cm^−1^ and very promising performance in the alkaline DMFC with peak power density of 40.2 mW cm^−2^.

Quaternized poly(phenylene oxide) (QPPO), where one of the polymers has simple aromatic structures, was used to develop AEMs for fuel cells. Cheng et al. [185] reported the incorporation of quaternized CNC (QCNC) into the QPPO polymer matrix for the first time. Different proportions of the QCNC (1–4 wt%) to QPPO were used to enhance the overall AEM properties. The obtained IEC for the QPPO/QCNC-2 membrane was 1.04 meq g^−1^, with a significant improvement in -OH^−^ conductivity that reached 28 ± 0.7 mS/cm. The membrane retained a conductivity of 5.0 mS cm^−1^ under accelerated degradation treatment at 80 °C in 1 mol/L NaOH for 120 h. The performance of the fuel cell’s attained peak power density of 392 mW cm^−2^ at 60 °C without back pressure for QPPO/QCNC-2 membrane compared to 270 mW cm^−2^ for pure QPPO, and at 60 °C without back pressure.

To enhance the performance of cellulose-containing QPPO membranes, quaternized CNF (QCNF) was combined with quaternized GO (QGO) as fillers in the QPPO matrix, which was crosslinked to form QPPO/QCNF/QGO composite membranes by Das et al. [186]. The obtained QPPO/QCF/QGO membrane with a compositional ratio of 100:1:1 attained the highest IEC of 2.64 meq g^−1^ and -OH^−^ conductivity of 114.64 mS cm^−1^ at 25 °C, suggesting potential successful applications in AEMFC. Earlier, Das et al. [187] used another approach involving crosslinking of -OH^−^-conducting 1,4-diazabicyclo [2.2.2]-octane (DABCO)-cellulose nanofiber (CNF) with DABCO–PS using 1,4-dibromo butane. The composite membranes (DABCO–CNF/DABCO–PS) showed conductivity in the range of ca. 39–74 mS cm^−1^ at 25 °C, depending on the ratio, and reaching 128 mS cm^−1^ at 80 °C, derived from the nanophase separation and densely distributed ionic channels. Such a strategy provides a valuable prospect to design a robust AEM.

Quaternary-ammonium PEEK (QAPEEK) was used as a primary polymer for the fabrication of the composite AEM membrane that was filled with sulfonated CNF, as reported by Peng et al. [188]. The membrane was thin and exhibited high conductivity but suffered excessive water swelling, which compromised the hydromechanical properties. The content of S-CNF was varied to avoid this problem without affecting ion conductivity. The QAPEEK-8/S-CNF-1 is considered an optimum membrane that achieved ionic conductivity of 21.4 mS cm^−1^ at 30 °C, and the fuel cell performance of the QAPEEK-8/S-CNF-1 membrane achieved a power density of 930 mW cm^−2^ compared to pure QAPEEK, which was 760 mW cm^−2^.

A different strategy was used for the fabrication of AEM in a previous study by Jaohuar et al. [53]. These researchers developed a CA-based radiation-grafted AEM. The vinylbenzyl chloride (VBC) monomer was radiation grafted onto CA using gamma radiation, leading to the CA-*g*-PVBC copolymer, followed by functionalization with trimethylamine (TMA). The obtained membrane, CA-*g*-PVBC/TMA, demonstrated an ionic conductivity of 16.3 mS cm^−1^ that has the potential for application in AEMFC. A high-performing synthetic AEM based on semi-interpenetrating polymer networks (SIPNs) was fabricated using CA nanofibers, and cross-linked chitosan (CS) was fabricated by Samaniego et al. [189]. The procedure involved deacetylation of cellulose diacetate (CDA) nanofibers into cellulose monoacetate (CMA), and cellulose improved the IEC by up to 12% due to the conversion of the acetyl group into hydroxide groups, providing active sites for functionalization. The 150 μm cellulose-chitosan SIPN AEM demonstrated maximum ionic conductivity of 21 mS cm^−1^ at 60 °C.

A series of nanocomposites consisting of quaternized PVA and NC was used as the AEM for a direct alcohol-hydrogen peroxide fuel cell (DAHPFC) application operating under alkaline conditions [190]. PVA and NC were quaternized separately with hexadecyltrimethyl ammonium bromide (HDT) and glycidyltrimethyl ammonium chloride (GAC), cross-linked, and cast to achieve quaternized polyvinyl alcohol/quaternized nanocellulose (QPVA/QNC) membranes upon thermal treatment. It was observed that QPVA/QNC/GAC30% membranes possess a maximum ion conductivity of 9.85  ±  0.07 mS cm^−1^ at room temperature and 29.07  ±  1.76 mS cm^−1^ at 80 °C with an IEC of approximately 1.14 meq g^−1^. The addition of QNC was found to enhance the alkaline stability of the optimized QPVA/QNC membrane with fewer ion conductivity losses.

Wang et al. [191] reported a new strategy of simultaneous crosslinking and filling using functionalized BC as a dual-function porous support and filler, and brominated poly (phenylene oxide) (Br-PPO) as a crosslinking agent and filler. The obtained quaternized-PPO (QPPO)-filled PBC composite membrane showed an extremely low swelling ratio and improved mechanical properties. Moreover, the membrane exhibited a conductivity of hydroxide ions of 62.58 mS cm^−1^, which was considerably higher than that of the pure QPPO membrane by up to 236.0% (at 80 °C). The successful fabrication of the PBC3/QPPO membrane provides an effective solution for breaking the trade-off effect through a dual-functional method. A summary of previous research studies on cellulose-based membranes for AEMFC is presented in Table 5.

Based on the various AEM fabrication strategies discussed earlier, the different forms of cellulose materials introduced to several polymer matrices have improved the AEMFC properties and their performance. The choice of modification method should be carefully considered based on the specific requirements of the fuel cell application, aiming to balance these properties for achieving optimal performance. However, the performance of cellulose-based membranes critical properties, such as ion conductivity, mechanical strength, and gas permeability, must be further improved to ensure long lifetime in AEMFC. Optimization of the composition of the membranes and the content of cellulosic components is necessary to obtain robust AEMs. The stability of the membranes in alkaline conditions was not evaluated in many previous studies. Thus, addressing the stability of the membranes via the accelerating degradation test has been an essential part of membrane development. To avoid decreasing the mechanical stability after adding the functional groups and enhancing the chemical stability, the crosslinking of the cellulose with other polymeric materials in the membrane matrix provides a reasonable solution, but the level of crosslinking has to be optimized. Moreover, there are other possibilities for cellulose-based membranes to be integrated with other types of organic and inorganic materials to enhance their properties and performance. 

## 7. Batteries

Batteries play a pivotal role in modern energy storage, serving applications that range from portable electronics to grid-scale renewable integration. Among them, LIBs dominate the portable and electric mobility markets due to their high energy density, efficiency, and relatively low self-discharge. In contrast, VRFBs have emerged as a preferred choice for large-scale energy storage, offering long cycle life, inherent safety, and flexible scalability. In both systems, the membrane is a key component, serving as a separator in LIBs and as an IEM in VRFBs, directly influencing their performance, efficiency, and safety. The recent advances in cellulose-based membranes for LIBs and VRFBs, highlighting their fabrication methods, functional modifications, and potential to address the specific challenges of each technology, are reviewed in the next section.

### 7.1. Lithium-Ion Batteries

In LIBs, the separator serves as an insulating layer between the cathode and anode, playing a vital role in device safety. It physically blocks direct electrical contact between electrodes to avoid short circuits, while providing mechanical support [192]. At the same time, it must allow lithium-ion transport during charge–discharge cycles while preventing electron flow [193]. The separator also acts as an electrolyte reservoir, supporting efficient ion transport and maintaining ionic conductivity [194]. Despite these functions, safety concerns persist, as conventional separators have low melting points and can shrink or melt under high temperatures or mechanical impact, causing electrode contact and potentially leading to thermal runaway, fires, or explosions [195].

For LIBs to operate efficiently and safely, the separator must meet several key criteria. It should be chemically and electrochemically stable with both the electrolyte and electrodes, without degrading over time. A thin membrane (~25 µm) is preferred, as thicker membranes increase resistance to lithium-ion transport and reduce energy density due to dead volume. The separator should ensure fast ionic transport while maintaining mechanical and thermal integrity, enabling long-term cycling stability with high-capacity retention. Thermal shrinkage should be minimal, ideally less than 5% after 60 min at 90 °C [196]. Polyolefins such as polyethylene (PE) and polypropylene (PP) are widely used due to their high mechanical strength and chemical stability [197,198]. However, they suffer from significant thermal shrinkage and melting at their respective melting points (135 °C for PE, 165 °C for PP), which can cause electrode contact and trigger safety hazards [199]. Their hydrophobicity also limits electrolyte wettability. In contrast, cellulose and its derivatives offer high thermal stability, excellent mechanical properties, hydrophilicity, and renewability, attributes that align well with the requirements of LIB separators and support stable cycling performance at high-capacity retention [35]. Extensive research has been dedicated to enhancing the properties of cellulose-based membranes for LIB applications. Although the hygroscopic nature of cellulose was initially viewed as a drawback due to its tendency to absorb moisture, it is now considered advantageous in developing membranes with controlled water uptake and potentially beneficial in improving ionic conductivity and overall battery efficiency [40].

Safety remains a major concern for LIBs, and cellulose-based membranes have demonstrated excellent thermal stability, often retaining structural integrity up to 160 °C or higher. BC-derived separators have gained attention for both LIBs and LISBs, as highlighted by Song et al. [200]. Jiang et al. [201] reported a BC nanofibrous membrane with no shrinkage up to 180 °C, and such behavior was attributed to its cross-linked 3D network. Despite being significantly thinner than the desirable thickness for LIBs, these 13 µm-thick membranes delivered a competitive specific discharge capacity (150 mAh g^−1^) compared to Celgard^®^ 2325 (141 mAh g^−1^) and showed no overcharge phenomena during LiFePO_4_/Li half-cell cycling.

An innovative approach to the fabric membrane for LIBs involves the incorporation of phytic acid, a bio-based flame retardant, into a BC framework with a zinc alginate binder and Zn^2+^–phytate coordination complex nanoparticles (BZP). This membrane retained 86% capacity over 1600 cycles and operated across a wide temperature range (−40 °C to 80 °C) with low flammability [202].

In another strategy, a BC-based composite membrane reinforced with a redox-active polypyrrole (PPy) layer was reported [203]. The membrane achieved a discharge capacity of 119 mAh g^−1^, which is over 1.5 times higher than conventional polyolefin membranes at a 2 C rate.

On the other hand, chitosan (CS) has also been shown to further improve the pore structure and porosity, which are essential for LIB applications, as advocated by Cheng et al. [204]. The chitosan was grafted onto BC (referred to as OBCS) via a chemical reaction. With the improved pore structure, the membrane achieved a high electrolyte uptake of 385%, indicating enhanced lithium-ion transport. The capacity retention of the battery remained at 90% after 100 cycles, suggesting good electrochemical performance.

Many other materials have also been incorporated with BC for LIB applications, leading to improved electrochemical properties and stability. Examples include ZrO_2_ [205], polyether block amide (PEBAX) [206], Al_2_O_3_ [207], zeolite imidazolate framework-8 (ZIF-8) [208], aramid nanofiber (ANFs) [209], halloysite nanotubes (HNTs) [210], 2,2,6,6-tetramethylpiperidine-1-oxyl radical (TEMPO) [211], and polydopamine (PDA) [212], among others.

Other than BC-based composites, CNCs have also been investigated for their potential application in LIBs. A study was conducted using CNCs prepared through facile self-assembly, resulting in free-standing meso-macroporous (mCNC) membranes, which was transformed into a lithium-ion-conducting membrane upon lithiation of the –OSO_3_H groups on the surface of the CNCs [213]. This membrane was thicker (75 μm) than most commonly reported membrane thicknesses for LIBs. Upon testing, it showed good lithium-ion conductivity of 2.0 mS cm^−1^ and a capacity retention of 93% after 100 cycles at a 0.5 °C rate. Another mesoporous CNC (mCNC) prepared via the same method was also reported by the same group, showing similarly promising results [214].

CNCs can also be incorporated with other materials to form composite membranes suitable for LIBs due to the presence of hydroxyl groups in CNCs, which render them more compatible with electrolytes. In a study by Zhou et al. [215], the CNCs were extracted from *sisal* through phosphoric acid hydrolysis to produce uniform nanocrystals, which were then combined with polyacrylonitryl (PAN) via electrospinning to fabricate a composite membrane. The resulting membrane delivered excellent electrochemical performance, achieving capacity retention of 97.4% after 100 cycles. At 200 °C, the CNC/PAN composite membrane exhibited a shrinkage of only 7.3%, in contrast to the PP separator, which began curling and softening at 120 °C and eventually underwent complete deformation. These results suggest that the superior thermal dimensional stability is primarily attributable to the PAN substrate, with CNCs providing additional reinforcement [215].

CNCs can also be incorporated with other synthetic polymers and metal oxides, such as PVDF [46] and polyacrylamide/titanium dioxide (PAM/TiO_2_) [216]. The incorporation of CNCs with these materials combines the intrinsic mechanical strength and thermal stability with the complementary properties of the host matrix, imparting ion transport properties yielding membranes with enhanced overall performance in LIBs.

CNF has been explored both as a standalone material and in combination with other components to enhance the performance of LIB separators. In one of the earliest studies, Chun et al. [217] examined the effect of varying the isopropyl alcohol (IPA)/water ratio in the dispersion medium on the pore size and porosity of pure CNF separators, later referred to as CNP. The best performance was obtained with 100% IPA, achieving an ionic conductivity of 0.77 mS cm^−1^ and 87% capacity retention after 100 cycles.

In a subsequent study [218], IPA was replaced with ethanol, producing an ultra-light, ultra-thin (12 µm) membrane (ECM) with slightly lower performance than CNP. Building on this work, Wang et al. [219] used tert-butyl alcohol (TBA) as the dispersion medium to fabricate CNF separators via a simple filtration method. Owing to its relatively low polarity, TBA reduced nanofiber aggregation more effectively than ethanol or IPA, yielding a more open and porous structure. This resulted in a substantially higher ionic conductivity of 1.90 mS cm^−1^, far exceeding IPA-based (0.77 mS cm^−1^) and ethanol-based (0.22 mS cm^−1^) CNF membranes, while maintaining excellent thermal stability with no shrinkage even at 160 °C. These findings confirm the strong potential of pure CNF as a high-performance standalone separator for LIBs and open opportunities for further enhancement through composite modification strategies.

Beyond a standalone separator, CNF composites incorporating polymers such as PVDF [220], PAN [221], and polyethylene glycol (PEG) [222] have been developed to further enhance separator performance. For example, PEG/CNF membranes exhibit superior lithium-ion transport due to high porosity and abundant oxygen-containing functional groups, resulting in improved initial capacity and capacity retention. While a reference PP membrane retained only 25% of its capacity, the PEG/CNF membrane maintained 80% after 300 cycles, demonstrating the effectiveness of composite modification strategies.

A study to mimic the commercial Celgard 2325 (PP/PE/PP) separator was carried out by Pan et al. [223] in which the group used CNFs extracted from *Cladophora* green algae to fabricate a tri-layer CNFs/PE/CNFs (CPC) membrane. Since PE is highly hydrophobic, the PE surface was rendered hydrophilic via O_2_/N_2_ plasma treatment for 30 s at 50 W. This treatment enabled strong lamination between the CNFs and PE through enhanced intermolecular interactions with the treated PE surface. Using this strategy, the resulting CPC membrane showed improved wettability compared to pure PE, leading to a higher ionic conductivity of 0.22 mS cm^−1^ compared to 0.16 mS cm^−1^ for PE. In cycling tests, the CPC separator achieved a capacity retention of 97.5% after 65 cycles, slightly outperforming pure PE (95.2%). Postmortem SEM showed that CPC led to more uniform Li deposition, reducing pitting and helping suppress dendrite growth, which is expected to improve cycle life.

In another study, PP nanobelts (PPNBs) were used as a structural skeleton to reinforce cellulose (PPNBs/CS) and mitigate shrinkage during dehydration [224]. The resulting membrane formed a sandwich-like structure, which delivered a superior ionic conductivity of 1.04 mS cm^−1^, as the three-layer configuration provided additional space for electrolyte absorption. The membrane also demonstrated excellent thermal stability, maintaining integrity at temperatures above 200 °C, with only 30% shrinkage from its original dimensions. In LIB tests, the PPNBs/cellulose composite separator achieved a high-capacity retention of 92% after 150 cycles, significantly higher than that of commercial Celgard 2400 (78%). A summary of previous research studies on cellulose-based membranes for LIBs is presented in Table 6.

From previous discussion, it can be observed that cellulose and its derivatives have demonstrated strong potential as next-generation separators for LIBs due to their intrinsic thermal stability, mechanical strength, electrolyte wettability, and environmental sustainability. Cellulosic forms such BC, CNCs, and CNFs have been used for the fabrication of cellulose-derived membranes and have shown superior performance compared to conventional polyolefin separators, with reduced thermal shrinkage, enhanced ionic conductivity, and in some cases, improved cycling stability and capacity retention. Composite membrane fabrication strategies, incorporating inorganic fillers, polymers, or flame-retardant additives, further expanded the functional window of cellulose-based membranes, enabling high performance even under elevated temperature or high-rate cycling conditions.

Nevertheless, several challenges must be addressed before large-scale application can be realized. These include achieving thin yet mechanically robust membranes comparable to commercial separators, precisely controlling porosity and pore size to balance ion transport with strength, ensuring chemical stability in liquid electrolytes, and developing scalable, cost-effective processing routes. Moreover, standardized benchmarking efforts remain limited, making it difficult to fully evaluate the new membranes industrial viability and sustainability. With continued progress in scalable fabrication and chemical modification strategies, cellulose-based membranes hold strong promise as safe and sustainable alternatives to conventional LIB separators.

### 7.2. Vanadium Redox Flow Batteries

To date, VRFBs have been deployed not only for large-scale industrial applications but also for residential power backup systems, as well as electric vehicle (EV) charging stations [225,226]. Their appeal lies in their flexible scalability, high operational safety, and long life, making them a promising technology for both off-grid and decentralized applications [227]. A critical component that governs VRFB performance is the membrane, which serves to separate the catholyte and anolyte, preventing the cross-mixing of vanadium ions in different oxidation states, while allowing the transport of protons to facilitate efficient charging and discharging. The overall energy efficiency (EE) of a VRFB is determined by its voltage efficiency (VE) and coulombic efficiency (CE), where VE is influenced by the proton conductivity and CE is governed by vanadium ion permeability [228].

In VRFBs, Nafion remains the most widely used due to its excellent chemical stability and high proton conductivity. However, it suffers from a significant drawback, which is high vanadium permeability, arising from the negatively charged SO_3_^−^ groups in its structure, which facilitate the transport of positively charged vanadium species [229,230]. In addition, the size discrepancy between vanadium ions (~0.6 nm) and the ionic domains in Nafion membranes (3–5 nm) further contributes to substantial vanadium permeability, significantly reducing battery performance [231]. In VRFB operation, the electrolyte typically contains < 3.0 M sulfuric acid [232], and strongly oxidizing VO_2_^+^ species are present during charging. As a result, membranes must maintain both chemical and mechanical stability under highly acidic and oxidative conditions to ensure long-term operation. From a techno-economic perspective, the high cost of Nafion, which can account for up to 40% of the total system, further motivates the search for cost-effective alternative membrane materials [233]. Other than Nafion, polybenzimidazole (PBI) has also been identified as possessing a promising membrane material for VRFBs as it has strong chemical resistance to acidic and oxidative environments [108]. Strategies to reduce Nafion content and enhance its performance, such as spray coating it onto the surface of PBI film to form Nafion/PBI (acid–base pairs) composite membranes, have improved the vanadium barrier properties compared to pristine Nafion membrane, but are still far from achieving satisfactory overall battery performance [234,235].

In recent years, cellulose-based materials have gained attention as a promising low-cost alternative to current materials’ components in VRFBs. In addition to being derived from abundant renewable resources, cellulose offers good chemical stability and tunable structural properties for this application. Several studies have reported the use of cellulose for the fabrication of IEM for VRFBs with improved properties. Mukhopadhyay et al. [236] designed a chemically stable CEM with high ion-selectivity by incorporating proton-conducting CNCs into a semicrystalline, hydrophobic poly (vinylidene fluoride-co-hexafluoropropylene) (PVDF-HFP) matrix. The hydrophobic PVDF-HFP phase suppressed electrolyte crossover, while CNCs, derived from low-cost wood sources, provided high proton conductivity due to abundant hydroxyl (-OH) groups, sulfonic acid (-SO_3_^−^) groups, and a robust intramolecular hydrogen-bonding network. The CNCs, with their high crystallinity (~86%), also exhibited high chemical and mechanical stability in strongly acidic electrolytes. The resulting CNC/PVDF-HFP membrane achieved a proton conductivity of 16 mS cm^−1^, a CE of 98.2%, and an EE of 88.2%, surpassing Nafion 115 with stable cycling over 650 cycles at 100 mA cm^−2^. Following this work, the same research group explored BC as a scaffold material to leverage its naturally interconnected 3D nanofiber network to enhance proton conductivity [237]. The obtained membrane achieved a proton conductivity of 9.5 mS cm^−1^, a CE of 97.6%, and an EE of 79.5% over 300 cycles at 100 mA cm^−2^. Under comparable conditions, its performance was lower than that of the CNC/PVDF-HFP membrane. While vanadium permeation was lower than Nafion 115, the data were presented as vanadium concentrations after 24 h rather than as calculated permeability coefficients, limiting direct comparison.

Inspired by the previous study, Mukherjee et al. [238] developed an ultrathin (20 μm) paper-based cellulosic IEM using an industry-friendly roll-to-roll process to impregnate CNCs into a paper scaffold with a PVDF-HFP binder. The membrane’s highest proton conductivity achieved was 6.9 mS cm^−1^ with good water uptake of 37–48%, comparable to Nafion (38%). However, this study primarily focused on fabrication and physicochemical characterization rather than electrochemical evaluation in a working VRFB. Therefore, further investigation is necessary to verify the membrane’s performance under different operational conditions.

Recently, another group developed a hybrid PEM by incorporating sulfonated cellulose nanocrystal (SCNC)/MXene hybrids into a PVDF-HFP matrix [239]. The SCNC/MXene synergy yielded low vanadium permeability (4.92 × 10^−9^ cm^2^ min^−1^), high proton conductivity (15.8 mS cm^−1^), and ion selectivity (3.21 × 10^6^ S min cm^−3^). The EE ranged from 81.6% to 90.7% over 40–120 mA cm^−2^, outperforming Nafion 212. The membrane was found to be 20 times cheaper than Nafion 212.

Other than using cellulose-based material, the modification of existing commercial membranes, such as Nafion, with CNC layers was also investigated. In the work carried out by Kim et al. [240], Nafion 212 was coated with polydiallyldimethylammonium chloride (PDDA) and CNC by the layer-by-layer (LbL) technique. The incorporation of PDDA/CNC bilayers has been shown to effectively suppress vanadium ion permeation, with performance improving as the number of bilayers increased. The optimal configuration, Nafion-[PDDA/CNC]_20_, had a total thickness of approximately 50.98 µm and achieved an EE of 89 % at 60 mAcm^−2^, comparable to Nafion 212. Self-discharge testing further confirmed its reduced vanadium crossover, as it maintained an open-circuit voltage (OCV) for about 74 h compared to 28 h for pristine Nafion 212. No further quantitative data on proton conductivity or vanadium permeability coefficients were reported.

Similarly, a BC membrane reinforced with a PFSA ionomer (BC–PFSA) was fabricated and exhibited an ionic conductivity as high as 60.5 mS cm^−1^, which is about 12.1 times higher than a standalone BC (5.0 mS cm^−1^) membrane [241]. Although the performance of BC–PFSA was slightly lower than Nafion 212, this strategy remains promising as it could help lower the cost of Nafion-based membranes.

Apart from surface modification and blending, the incorporation of inorganic fillers such as silica, SiO_2_, into nanocellulose has also been investigated to enhance both membrane robustness and ion selectivity to balance high proton conductivity and vanadium permeability. In the work carried out by Zhang et al. [242], a dense silica layer was formed on the surface of nanocellulose fibers via an in situ sol–gel method, producing core–shell-structured CNCs. These CNCs were then incorporated into an SPES matrix to prepare CNC-SPES hybrid membranes. When compared with pristine nanocellulose-reinforced SPES (NC-SPES) and Nafion 212, the CNC-SPES membrane exhibited superior mechanical strength (54.5 MPa) and maintained a high energy efficiency above 82% at 100 mA cm^−2^ over 200 charge–discharge cycles. The presence of the silica shell not only improved the structural integrity of the membrane but also suppressed vanadium ion crossover without significantly sacrificing proton conductivity. Although the ion selectivity of the CNC–SPES (7.94 × 10^4^ S min cm^−3^) was lower than that of Nafion 212 (9.9 × 10^4^ S min cm^−3^), it was still slightly higher than that of the NC–SPES (5.79 × 10^4^ S min cm^−3^).

For VRFB applications, the studies reviewed so far confirm the great potential of cellulose as a membrane material. Nevertheless, research on cellulose-based membranes for VRFBs remains limited, with only a handful of studies reported to date. The membranes fabricated through chemical modification are still limited, despite its suitability to address the requirement of highly acidic and oxidizing electrolytes operating medium in VRFBs. Exploring chemically modified cellulose could therefore be valuable for assessing its true feasibility under such harsh conditions. Furthermore, the cycling performance of cellulose-based membranes in realistic operating environments remains a mystery, with insufficient investigations. In fact, the highest reported cycle life to date is only 650 cycles, and the majority of studies lack comparisons with the Nafion membranes. Table 7 summarizes previous studies on the cellulose-based membranes for application in VRFBs. Beyond VRFBs, progress in using cellulose-based membranes for other RFB applications remains limited. Notably, few studies have explored wood-derived nanocellulose [243] and sulfonated CNFs [244] in organic aqueous RFBs (AORFBs), finding that these membranes outperformed Nafion 115. While promising, such studies are still scarce, leaving substantial room for further exploration of RFBs.

Cellulose-based membranes represent a promising, low-cost, and sustainable alternative to conventional IEMs in VRFBs. Their tunable nanostructure, high proton conductivity, and reduced vanadium ion crossover, especially when combined with polymers or inorganic fillers, have already demonstrated competitive or superior performance compared to Nafion in laboratory-scale studies. Nonetheless, several key challenges remain. Long-term chemical stability must be assured not only under strongly acidic and oxidizing electrolytes but also across a wide temperature range and through rigorous ex situ chemical stability tests. Balancing high proton conductivity with low vanadium permeability, mitigating swelling from excess water uptake, and maintaining adequate mechanical strength under stack pressure also remain unresolved. In addition, most reported studies demonstrate stability for only a few hundred cycles, well below the >10,000 cycles expected in practical VRFB systems. Future work should therefore prioritize chemically stabilized or hybrid cellulose membranes, scalable roll-to-roll fabrication methods, and extended durability testing under realistic operating conditions.

## 8. Supercapacitors

The separator is a crucial component in supercapacitors to ensure free flow of ion transport throughout interlinked electrolyte-soaked structure and to prevent internal short circuit between anode and cathode [115]. Thus, obtaining a robust separator with the desired properties plays a pivotal drive in optimizing the overall efficiency and applicability of supercapacitors. Polyolefin-based films are widely used as separators in supercapacitors. However, such materials meet persistent challenges, including shrinkage at high temperatures, poor electrolyte wettability, low mechanical strength, and non-renewable nature, all of which impact the efficiency and safety of the device [245]. Alternative polymer-ceramic composite membranes such as PVDF and (Al_2_O_3_, SiO_2_) [246], as well as gel electrolytes from PVA host such as PVA-H_2_SO_4_ and PVA-H_3_PO_4_, have been recently used to enhance the electrochemical performance of supercapacitors [247,248]. However, there is still a parallel growing demand for green materials with stable chemical properties, excellent thermal stability, low electrical resistance, and high charge transfer capability. 

Cellulose-based separators offer alternative sustainable materials for developing new separators with attractive properties for supercapacitors. Various novel strategies of cellulose-based separators fabrication have been adopted to enhance the efficiency of supercapacitors [249]. The various research strategies are reviewed and discussed in the next section.

BC has been explored as an alternative material for supercapacitor separators. Several studies reported that BC exhibited a wide range of results and output depending on the type of modifications. Yao et al. [250] deposited Ppy on CNT and BC fibers (CNT/BC) through in situ oxidative polymerization. CNT was first introduced to BC to facilitate CNT aggregations and dispersion, hence improving its function. Subsequently, Ppy was distributed on the surface of the CNT/BC gel and created a layered core–shell structure with a large surface area. The assembled device produced high specific capacitance at 228 F g^−1^ at the current density of 0.5 A g^−1^ with excellent cycling retention ability. This was followed by a study that reported the use of in situ-polymerized Ppy in BC that was pre-functionalized with zeolitic imidazolate framework (ZIF-67) and/or polydopamine (PDA) using ferric chloride oxidant [251]. This led to conductive hierarchical composite membranes. Without pre-functionalizing the BC, Ppy/BC showed that nanochannels in the composite separator were completely blocked due to the numerous disordered nanorods of Ppy. However, the composite membrane produced was quite thick and had a similar specific capacitance of 209.09 F g^−1^ at the current density 1.82 A g^−1^ depending on Ppy mass (5.5 mg cm^−2^). Another BC-based separator was introduced by Yue et al. using surface-coating modification via in situ chemical oxidative polymerization [252]. In their study, BC was pre-functionalized with graphene (GN) to yield BC/GN nanofibers, where BC was employed as a dispersant for improving GN distribution. The resulting composite separator produced high ionic conductivity of 621 S cm^−1^ and capacitance of 444 F g^−1^ with 95.11% cycling stability after 5000 cycles.

Besides BC, other types of cellulose forms, such as MCCs, were also used in the search for robust separators for supercapacitors. The MCC was first dissolved in ionic liquid ([DMIm][(MeO)(H)PO_3_] and [BMIM][TFSI]) and crosslinked with N N′-methylenebisacrylamide (MBAA), while poly(2-hydroxyethyl methacrylate) (pHEMA) was formed via in situ free-radical polymerization [253]. The resulting C-pHEMA/C-p-cellulose interpenetrating networks displayed enhanced mechanical and functional properties. The formed ionomeric sheet (100 μm) had ionic conductivity of 2.6 mS cm^−1^ and capacitance of 22.4 F g^−1^ at 30 °C. Furthermore, both these two properties were boosted to 22.4 mS cm^−1^ and 174 F g^−1^ when tested under a high temperature (120 °C).

CNC and CNF were also frequently employed to fabricate separators for supercapacitors. Some studies reported blending GO with CNC at various weight ratios before being spun into fibers and reduced chemically with hydrioiodic acid (HI) [254]. The composite separator showed an excellent ionic conductivity of 64.7 S cm^−1^ with good specific capacitance of 123.2 F g^−1^. It was revealed that the nano-rod shapes of CNC did not coat the GO sheets when blended, hence maintaining the direct electron pathway that assisted the ion transport.

Another study incorporated CNC to stabilize CNTs, which were crosslinked with PVA and poly(acrylic acid) (PAA) to form the core material of the separator [255]. Subsequently, the blends were combined with polyaniline (PANI) through in situ polymerization to fabricate the separator. The modification and fabrication produced a thinner separator (40 μm) with high specific capacitance of 164.6 F g^−1^. However, the ionic conductivity was 4.4 mS cm^−1^, a decreased value from previous studies due to the non-conducting polymer matrix PVA and PAA, both of which acted as barriers to the CNT conductive network.

CNF on its own was proven to be reliable for the use of separators based on studies by Shah et al. [115] and Xu et al. [256]. The CNF separators were sourced from local resources (i.e., cheap and abundant), synthesized, and fabricated physically (vacuum filtration and solution casting). Both separators have similar outcomes, such as forming a thin separator, reasonable ionic conductivity, and excellent specific capacitance. Both approaches proved to work out greatly, despite their different sources, suggesting that CNFs are reliable and promising separators.

Moreover, various modifications and improvements have been made in CNF-based separators to enhance the output, such as hydrothermal deposition using manganese dioxide [257], a combination of ultrasonic dispersion and freeze-drying [258], vacuum filtration [259,260], carbonization, and in situ polymerization [261,262]. Modified CNFs for separators seem to yield quite thick separators with good ionic conductivity. Nonetheless, it was realized that these modifications improve the specific capacitance produced where all separators gave a high specific capacitance output ranging from 103 to 1144.3 F g^−1^. The separator made of CNF was transformed into TEMPO-oxidized cellulose nanofibers (TOCNFs), followed by hydrothermal deposition of MnO_2_ to help with the specific surface area, high aspect ratio, and tremendous carboxyl groups contained on the surface of CNF [257].

Meanwhile, the CNF was reinforced with carbon fiber (CF) before the dispersion of multiwall carbon nanotubes (MWCNTs) where the CNF can bind strongly to the carbon materials (CNF and CNT) and form porous 3D conductive network hybrid aerogels (CF-CNF/MWCNT-Has) [258]. CNF were also functionalized by MOFs as reported by Zhou et al. [259]. The MOF was nickel—2,3,6,7,10,11-hexahydroxytriphenylene (Ni-HITP) and the loaded CNF suspension was vacuum filtrated to produce CNF@Ni-HITP nanosheets, which demonstrated good ionic conductivity and capacitance. The separator was constructed by wrapping the CNF with Ni-HITP continuously, which formed conductive interconnected layers of nanofibers.

Another popular modification involving polypyrrole (Ppy) was carried out by in situ polymerization onto CNF and vapor-grown carbon fiber (VGCF) hybrid aerogel film [261]. The prepared CNF/VGCF/Ppy aerogels were used as electrodes for the supercapacitor, delivering a maximum value of 11.25 S cm^−1^. This was coupled with capacitances of 8.61 F cm^−2^ at 1 mV s^−1^ (specific area capacitance) and 678.66 F g^−1^ at 1.875 mA cm^−2^ (specific gravimetric capacitance) and retained >90% of its initial capacitance after 2000 cycles. Zhang et al. [262] used the CNFs to inhibit the GO agglomerating formed pore structure from their hydrophilic properties, thus improving ion access throughout the separators. The suspension was then coated with Ppy and formed aerogels denoted as CNFs/rGO/PPy. Meanwhile, Lv et al. [263] polymerized pyrrole (Py) on a suspension of CNF and CNTs and this led to an improvement of the capacitance and ionic conductivity in the hybrid sheet with these polymerization modifications, especially when CNF is dispersed in carbon fiber [261].

Another example of CNF was surface functionalized with CNTs and GO using the hydrothermal method, as reported by Ramesh et al. [264]. The formed Cell/MWCNT/rGO/Co_3_O_4_/SnO_2_ nanocomposite was tested as an electrode for supercapacitor applications. The capacitance was 215 F g^−1^ and 181 F g^−1^at a current density of 0.2 A g^−1^ and 0.4 A g^−1^, respectively. The electrode retained 88% capacitance after 1000 cycles.

**Figure 6:**
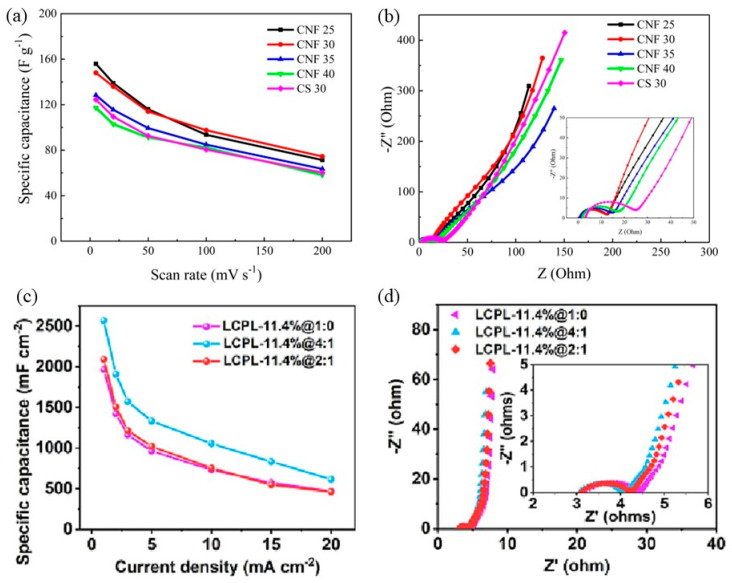
Properties of CNF-based membranes: (**a**) Specific capacitance with different thicknesses at different scanning rates, (**b**) Nyquist plots with different thicknesses [115], (**c**) specific capacitance at different current densities, and (**d**) Nyquist plots at different ratios of lignin and pyrrole [265]. Reprinted with permission from Shah et al. [115] and Jiran et al. [265].

Further studies on CNF as the best matrix for separator fabrication implemented surface functionalization of CNF with cationic poly(diallyldimethylammonium chloride) (PDADMAC) in the presence of sodium chloride [115]. PDADMAC increased the cationic surface charge of the CNFs, hence increasing the ionic conductivity and specific capacitance. The PDADMAC/CNF separator was only 30 µm thick with 5.0 mS cm^−1^, which produced 185.3 F g^−1^ at 0.1A g^−1^ with 100% capacitance retention and high efficiency for over 10,000 cycles (Figure 6a,b). Ramesh et al. [264] modified CNF through TEMPO-oxidation and in situ polymerization of pyrrole without fully removing lignin content in the cellulose pulp, hence forming sodium lignosulphonate/polypyrrole (LS/PPy) in lignin-containing cellulose nanofibrils (LCNFs). The (LS/PPy)/LCNFs separator exhibited a great specific capacitance of 197 F g^−1^ (Figure 6c,d).

Modified derivatives of cellulose such as methylcellulose (MC), carboxymethylcellulose (CMC), and sodium carboxymethylcellulose (NaCMC) have been also used in the fabrication of separators. Unlike cellulose, which contains only hydroxyl groups, it was replaced by methox- groups in its derivatives, which improved the cellulose stability in water and enhanced its physical and mechanical strength in the same way as some synthetic plastic polymers. 

In a study by Kim et al. [266], a lithium trifluoromethanesulfonate-methyl cellulose (LiTFS–LiSMC) polymer electrolyte was prepared by incorporating lithium tri-fluoromethanesulfonate into chemically modified methyl cellulose. The obtained electrolyte separator exhibited good conductivity of close to 1 mS cm^−1^. The 20 wt%LiTFS–LiSMC separator showed comparable specific capacitances to a standard liquid-electrolyte counterpart with an extraordinary stability of >20,000. This stability reflects the ability to undergo a large number of charge–discharge cycles. Another MC matrix was polymerized with (PANI) and loaded with copper sulfide (CuS) nanoparticles deposited on reduced graphene oxide (CuS@rGO), which were used to develop a separator by Mona et al. [267]. The MC/CuS@rGO separator displayed reasonable ionic conductivity (0.107 µS cm^−1^) and 100% capacitance retention for more than 10,000 cycles. Polyaniline (PANI) loaded with copper sulfide (CuS) nanoparticles deposited on reduced graphene oxide (CuS@rGO) was used to develop a separator by Mona et al. [267]. The MC/CuS@rGO separator displayed a reasonable ionic conductivity as high as 0.107 µS cm^−1^. It also extraordinarily demonstrated a 100% capacitance retention for more than 10,000 charge/discharge cycles. 

According to another study by Lee et al., CMC was subjected to ice-templating and subsequent carbonization to form a porous carbon network that was proposed as a separator [268]. The thermal treatment of CMC yielded a superior specific capacitance at 210 F g^−1^ at 1 A g^−1^ in a 6.0 M KOH electrolyte solution with excellent cycling stability of 100% at 10 A g^−1^ after 10,000 cycles. In addition, CMC was found to be quite compatible, allowing in situ copolymerization with poly(3,4-ethylenedioxythiophene)/polystyrene sulfonate into a chemically modified CMC/PEDOT/PSS [269]. The resulting separator, with a thickness of 98 μm, showed superior ionic conductivity at 45 S cm^−1^ and has specific capacitance of 116.4 F g^−1^ at a current density of 1 mA cm^−2^. This separator also maintained 93.4% retention capability even after 10,000 cycles, demonstrating a long lifetime.

In another study, NaCMC was reinforced by poly(3,4-ethylenedioxythiophene) (PEDOT) and aluminum oxide (Al_2_O_3_) microparticles, resulting in a separator with 0.6 mF g^−1^ specific capacitance, and the separator maintained a stability of 2500 cycles [270]. NaCMC can also be modified with pectin and glycerol plasticization and lithium salt incorporation, as reported by Riyadh Abdekadir et al. [271]. The solution-casting method was used to produce a rather thick separator (310 μm) that had ionic conductivity of 0.355 mS cm^−1^ and specific capacitance at 6.18 F g^−1^, obtained at a scan rate of 5mV s^−1^ in 2300 cycles. 

Another strategy to prepare a separator was to directly use a freestanding cellulose. For example, Zou et al. [272] used cellulose, reduced GO, and silver hybrid films to fabricate a separator through Tollen’s reduction and hydrazine treatment. The film was subsequently functionalized using successive ionic layer adsorption and reaction with iron (III) oxide (Fe_2_O_3_) particles and formed a freestanding Cell/RGO/Ag/Fe_2_O_3_. The separator had a thickness of about 620 μm and displayed commendable volumetric capacitance at 75.7 F cm^−3^. However, no ionic conductivity and specific capacitance were reported despite showing a good inconspicuous semi-circle in the Nyquist plot, which suggested the presence of a good conductivity of the film.

In another attempt to use free-standing cellulose, Salado et al. [273] assembled cellulose films that were coated sequentially with PDA or PANI and PPy though in situ polymerization that enhanced the hydrophilicity and conductivity of the cellulose/PDA/PPy separator. The film had a 134 μm thickness and exhibited an ionic conductivity as high as 0.59 S cm^−1^ and a maximum specific capacity of 32 F g^−1^ at a scan rate of 10mV s^−1^. The film also withstood 250 cycles and retained 96% of its initial capacity. 

Hsu et al. [274] also performed an in situ polymerization of PANI after the nanocellulose suspensions were blended with rGO to form an NC/RGO/PANI film. The thin separator (40 μm) showed a specific capacitance of 79.71 F g^−1^. Another separator was obtained using a suspension of nanocellulose and ZIF-67-derived porous Co_3_O_4_ polyhedron, forming a hybrid layer-by-layer film of the nanocellulose/polyhedron composite (NPC) [275]. The flexible NPC-60 film electrode obtained from the 60/30 ratio of porous Co_3_O_4_ polyhedron to NFC had a higher capacitance of 594.8 mF cm^−2^ at 5 mV s^−1^ in 6 M KOH compared to other samples.

Another study reported the fabrication of a hollow polypyrrole/cellulose hybrid hydrogel electrode from cellulose and PPy using the facile strategy of electrochemical deposition through the deposition of cellulose on a nickel matrix followed by PPy electrochemical deposition and an etching treatment [276]. The matrix had good conductivity at 0.606 S cm^−1^ with a high specific capacitance of 255 F g^−1^, in addition to a retention capability of 77% at a high current density of 30 A g^−1^. 

A cellulose derivative, namely hydroxypropyl cellulose, was also used to assemble an electrolyte in a study by Ko et al. [277]. These workers sulfonated and blended cellulose with SA via an epichlorohydrin-aminomethanesulfonic acid reaction and then combined with it aluminum fumarate to form a sulfonated cellulose-aluminum fumarate film (A520-SC). The film produced 135.14 F g^−1^ at 20 wt% of aluminum fumarate (A520) and 172 mS cm^−1^ ionic conductivity.

González et al. [278] studied another separator by incorporating various polythiophene-derivative polymers through spraying or casting onto a cellulose substrate. It was found that the highest ionic conductivity that can be achieved is 0.77 S cm^−1^ through in situ polymerization of CNF and the spray-coating technique.

Cellulose from bamboo pulp was also prepared as an aerogel as an economic and sustainable electrode in applications of supercapacitors by Yang et al. [279] The cellulose was carbonized to obtain activated carbon fiber aerogel (CFA). The CFA demonstrated a specific capacitance of 409 F g^−1^ at a 5 mV s^−1^ scan rate was carbonized at 1000 °C. The aerogel was found to have a retention rate of 82% at a scan rate of 200 mV s^−1^.

Wan et al. [280] also used bamboo fiber to fabricate cellulose-derived carbon aerogel (CDCA) that was ball-milled with iron oxychloride (FeOCl) and subsequently shaped it into electrodes. The electrode delivers a specific capacitance of 647 F g^−1^ at 2 mA cm^−2^ in a remarkable cycle stability with less than 10% capacitance loss after 10,000 cycles. 

Zhang et al. [281] developed all-CNF-made components for an asymmetric supercapacitor. Particularly, nanocellulose-derived matrices were further modified to be used as the cathode, the anode and the separator. The assembled system delivers a high capacitance of 64.83 F g^−1^ at 0.25 A g^−1^ and had high an ionic conductivity of 0.265 S cm^−1^.

Another study reported separator fabrication by dissolving cellulose followed by modification with ionic liquid, 1-allyl-3-methylimidazolium chloride (AMIM-Cl), before casting the mixture in a mold [282]. The resulting separator had a 150 μm thickness with 0.2986 S cm^−1^ ionic conductivity and 130 F g^−1^ specific capacitance of 0.5 A g^−1^ with high retention ability of 82% after 4000 discharge cycles. In a another study by Dang et al. [283], N, S dual-doped hierarchical porous carbon aerogels (NSHPAs) were fabricated by dissolving cellulose in a ternary solvent followed by gelling and carbonization. The obtained material with uniform heteroatom doping was proposed as a heteroatom-doped carbon electrode material. It was found that in 2M H_2_SO_4_, the separator exhibited good capacity at 329 F g^−1^ and a 0.5 A g^−1^ current density.

AMIM-C was also blended with regenerated cellulose and PVDF [114]. The blended mixture was converted to a separator through solution casting (RC@PVDF). The fabricated separator was a 20 μm thick film that had a conductivity of 3.87 μS cm^−1^ with 174 F g^−1^ specific capacitance at a current density of 4 A g^−1^ for 20,000 cycles and a retention capacity of 99.6%. Another regenerated cellulose was modified and regenerated with calcium carbonate and AMIM-Cl to form a cotton cellulose membrane (CCM) [284]. The obtained separator had 15 μm thickness with 16.32 mS cm^−1^ ionic conductivity. The separator exhibited 145.6 F g^−1^ specific capacity at 1 A g^−1^ current density. Cellulose from coffee waste showed potential as a separator extracted by simple alkali and bleach treatment [285].

The cellulose obtained from the coffee waste formed a CNF that was further modified into CNF membranes (CWCNF). The membrane separator had a 25 μm thickness with 3 mS cm^−1^ ionic conductivity and achieved a specific capacitance of 1768 F g^−1^ at a scanning rate of 5 mV s^−1^. A summary of previous studies on cellulose-based membranes for application in supercapacitors is presented in Table 8. 

As can be concluded from previous studies, cellulose-derived supercapacitor separators provide interesting alternative materials for supercapacitors because they are sustainable, highly wettable by electrolytes, and have good ionic conductivity. Nevertheless, such materials still face some practical barriers before full adoption. For example, the mechanical stability of cellulosic separators is often lower than that of polyolefin counterparts, which is most likely encountered when fabricating separators with thin thicknesses needed for high conductivity (low resistance). Moreover, the control over the pore size developed during the applied procedure remains as an issue since very large pores increase the risk of electrical short circuit, whereas too-small pore sizes retard ion transport. Furthermore, the presence of excessive hydrophilicity can cause excessive swelling in aqueous systems, leading to dimensional instability. Furthermore, the chemical stability and durability of cellulose-based separators in organic or ionic-liquid electrolytes have not been validated yet and require further investigations. Finally, most high-performance lab-scale cellulose-based separators rely on nanocellulose processing, surface functionalization, or nanoparticle coating, therefore not only increasing fabrication complexity cost and undermining scalability but also challenging reproducibility and consistent quality, thereby posing challenges to commercial deployment.

## 9. Reverse Electrodialysis

RED is a salinity gradient-harvesting technology that utilizes two different salt concentration solutions. RED is usually employed between freshwater river and saltwater sea connections where a potential gradient can be established to produce electricity. The system consists of stacks of IEMs in alternating patterns of cation and anion exchange membranes, which are filled with water of different salt concentrations. As these waters pass through the membranes, ions are separated based on their charges. Cations go through CEM, and anions permeate through AEM, which in turn creates ionic flux that generates electricity at the cathode and anode ends. The requirements needed for an IEM are high permselectivity, where the membrane should be highly permeable for the counter-ion but not co-ion, have a low degree of swelling when in contact with water so as to avoid the polymer dissolving and resistance increment, and lastly, show good mechanical strength and stability [286]. In addition, the IEM should have low electrical resistance to ensure less electrical energy is lost during ion transfer. However, the RED application still lacks customized membranes compared to other electrochemical systems. Efforts have been made to develop alternative IEMs using synthetic polymers in addition to cellulose-based membranes that have shown strong potential for application in RED.

The use of cellulosic-materials in the development of IEMs has grown significantly in the past decade [19,287,288]. For example, a bacterial cellulose-based membranes studied previously showed the credibility of BC as both a CEM and AEM for a RED system [289,290,291,292]. BC was favored in many studies because of its 3D structure, which makes up a large hierarchical structure and offers ample support to ion transport and ion selectivity. Wu and coworkers first developed a TEMPO-oxidized and etherified BC membrane without other fillers or a composite, assembling a negatively charged membrane (NBC) and a positively charged membrane (PBC) [289]. Both membranes had the same controlled thickness of 90 μm, which produced 1.0 and 0.42 mS cm^−1^ ion conductivity with 0.23 W m^−2^ output power density. This is proof that with surface functionalization of a pressed BC membrane, a low-cost, environmentally friendly membrane with scalability potential is possible.

Wu et al. [290] developed a negatively charged carbomethyl bacterial cellulose membrane (BC-CMC) and a positively charged chitosan quaternary ammonium bacterial cellulose membrane (BC-HACC) with thickness as low as 20 μm, which led to a power density of 2.25 and 0.45 W m^−2^ for BC-HACC and BC-CMC with only 0.78 mS cm^−1^ ion conductivity, respectively. The adopted method was successful in adjusting the surface charge density and size of nanochannels for efficient ion transport. Meanwhile, Sheng et al. [291] reported another BC-based membrane by dismantling and reconstructing the BC while adding GO and LDH to assemble negatively (NBC/NGO) and positively (NBC/PLDH) charged composite membranes, respectively. The obtained membranes were ultra-thin (7 μm), which demonstrated ionic conductivity of 0.52 to 0.64 mS cm^−1^ and an output power density of 0.70 W m^−2^.

**Figure 7:**
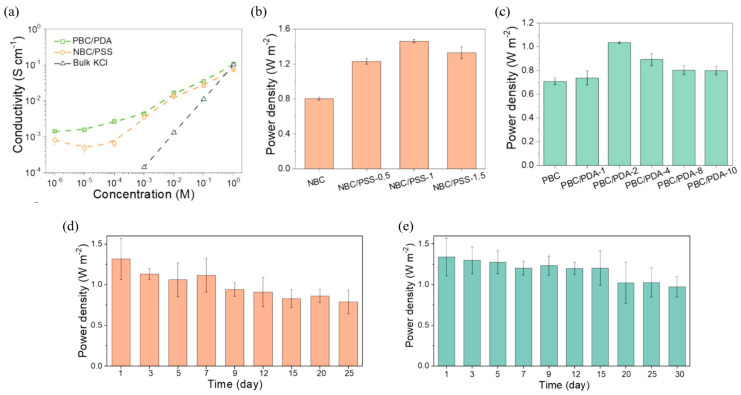
Properties of BC-based membranes: (**a**) Ionic conductivity of composite membrane NBC/PSS−1 and PBC/PDA−2 in different KCl concentrations. Power density of (**b**) NBC/PSS membrane at different styrenesulfonate concentrations and (**c**) PBCPDA at different dopamine concentrations. Stability of (**d**) NBC/PSS−1 and (**e**) PBC/PDA−2 membranes under 50-fold concentration gradients throughout 30 days. Reprinted with permission from Zhouyue et al. [292].

A study by Zhouyue et al. [292] demonstrated a remarkable increase in membrane thickness to 160 μm with a rather high ion conductivity, reaching 1.4 and 0.8 mS cm^−1^ for their positively and negatively charged membranes at 2 g L^−1^ dopamine and 1 mol L^−1^ styrenesulfonate concentrations, respectively (Figure 7a). Their method involved using TEMPO-oxidized and quaternized BC initially before polymerizing with poly (sodium p-styrenesulfonate) (NBC/PSS) to make a negatively charged bacterial cellulose membrane and poly(dopamine)(PBC/PDA) to yield a positively charged bacterial cellulose membranes, respectively. The membranes further produced 0.99 and 1.14 W m^−2^ output power density when tested with different concentration gradients of artificial seawater and freshwater (Figure 7b,c). When further studied, 50% of the concentration gradients exhibited power density above 0.79 (NBC/PSSA-1) and 0.98 W m^−2^ (PBC/PDA-2) after continuous testing for 30 days (Figure 7d,e).

Besides BC, other nanocellulose materials, such as CNF and CNC, have also received significant attention for the fabrication of IEMs for RED. For an example, Sun et al. [293] fabricated CNF sonicated with MXene nanosheets (MXene/CNF) through vacuum filtration (physical fabrication) and obtained a 46 μm-thick membrane with a stable and high ion conductivity at 4 mS cm^−1^ under a low-concentration gradient. The membrane showed excellent 2D structured nanochannels that improved the ion transport, whereby under a 1000-fold salt concentration gradient, the membrane generated a power density of 0.15 W m^−2^, which reflected the potential for applications in harvesting osmotic energy. A similar physical membrane fabrication method reported the use of GO as a filler with a CNF substrate [294]. The obtained membrane was ultra-thin, with a thickness of 9 μm that allowed a remarkable ion conductivity (0.135 S cm^−1^) leading to 4.19 W m^−2^ output power density at room temperature. The power density was also reported to be boosted when the temperature increased to 50 °C. The study proved that CNFs constitute a promising membrane matrix, with their easily achieved fabrication and modification.

Gao et al. [295] used another carbon-based filler, i.e., graphitic carbon nitride (g-C_3_N_4_) with a CNF substrate, using the same physical fabrication method that produced a membrane with a 2D lamellar nanochannel structure. The resulting membrane supplied 0.15 W m^−2^ power density with only 14 mS cm^−1^ ion conductivity, which was further increased to 0.22 W m^−2^ when the temperature increased to 60 °C. The g-C_3_N_4_/CNF membrane exhibited prolonged stability for 30 days, which confirmed the overall superiority of carbon-based fillers in cellulose nanofibers-based membranes. 

Li et al. [296] managed to fabricate another ultra-thin membrane (6.76 μm) from CNTs and CNF using a blending method. The membrane produced 5.53 W m^−2^ of output power density using saline and freshwater gradients, which was further increased to 6.51 W m^−2^ when real seawater and freshwater were used. Such interesting results emphasized the significance of the formation of the 2D nanochannel network when blending CNTs into CNFs. On the other hand, Luo et al. [297] reported the use of zero-dimensional material such as carbon quantum dots (CQDs) as a layered filler with CNTs (one-dimensional) and GO (two-dimensional) into the CNF substrate through vacuum filtration. The formed free-standing membranes with loaded layers could convert light to heat and build heat gradients across the membrane, which improved the ion transfer efficiency through the composite membranes. This led to an output power density of 3.55 W m^−2^ at 25 °C that was increased to 7.67 W m^−2^ output power density when the temperature increased to 53 °C.

In another approach, CNF was mixed with butane 1,2,3,4 tetracarboxylic acid (BTCA) (cross linker) and sodium hypophosphite (SHP) forming suspension that was converted to free standing and flexible membranes by solution casting and subsequent heat treatment at 150 °C [298]. The reaction introduced one to three carboxyl groups per BTCA molecule to the backbone of the CNF, increasing the negative charges to transport the cations along the CNF chains. The ionic conductivity of this low-cost membrane (CNF/BTCA/SHP) reached 8.1 mS cm^−1^ in 0.1 M KCl under pH 8.1 at 20 wt% BTCA. Such properties make this membrane of potential for RED applications.

CNF can also be used as a substrate for both CEM and AEM, with 2,2,6,6-tetramethylpiperidine 1-oxy radical (TEMPO) oxidation and etherification modification forming suspensions, being vacuum filtered to a 40 μm-thick membrane using a polycarbonate membrane with a pore size of 0.1 μm (T-CNF and E-CNF) [299]. Negatively charged carboxyl groups and positively charged quaternary ammonium groups in the cellulose nanofibers were obtained by converting the hydroxyl groups through oxidation and etherification, resulting in 17 and 16 mS cm^−1^ ion conductivity, respectively. The membrane also produced a maximum output power density of 2.87 W m^−2^ when the external load reached 20kΩ, and the power continued to be generated at the same rate for a month, which indicated an excellent membrane stability. 

Another CEM was molded using CNF extracted from a bamboo by milling [33]. The DMAC-treated CNF was dissolved in ionic liquid of 1-Allyl-3-methylimidazole chloride to produce polymer solutions that were cast on a glass plate and immersed in deionized water. The obtained thin membrane obtained after drying displayed an ionic conductivity of 0.364 mS cm^−1^ coupled with an excellent 2.2 W m^−2^ power density at an only 50-fold salinity gradient for continuous 60-day tests, demonstrating promising membrane stability. 

A study by Lin et al. [300] reported the fabrication of a functionalized *Cladophora* nanocellulose membrane that was extracted from algae with thermo-responsive ability by atom-transfer radical polymerization (ATRP) of poly(N-isopropylacrylamide) (PNIPAM). The CNF suspension was then vacuum filtered and formed a 50 μm-thick membrane (C-CNF). The membrane gained the ability to achieve a low critical solution temperature at 37 °C, and maximum power density of 10.1 W m^−2^ in RED cells under a 50-fold concentration gradient at 50 °C. 

An ionized wood-based IEMs were fabricated in forms of CEM and AEM for the first time [301]. The wood membranes have nanochannels sustained by the natural CNF by cutting the wood pieces perpendicular to the growth direction of the tree. Then, the wood pieces are converted to positively and negatively charged membranes through direct surface functionalization, which is etherification and TEMPO oxidation modification (P-Wood and N-Wood). The ionic conductivity obtained was quite remarkable and as high as 0.2 mS cm^−1^ for AEM and 0.4 mS cm^−1^ for CEM, despite the thickness of both membranes, with 0.00514 W m^−2^ as the maximum output power density. Since the membrane is made up of purely low-cost basswood, these membranes offer an inexpensive and naturally abundant solution for development of sustainable IEMs. 

Other RED membrane development efforts involved the use of a porous (conical shape) polyethylene terephthalate (PET) substrate to incorporate TEMPO-oxidized cellulose nanofibers (TOCNFs), as reported by Xu et al. [302]. The abundance of carboxyl and hydroxyl groups in TOCNs regulated the ion transport of the membranes while PET provided the nanofludic current gate for the composite membrane to increase the efficiency in cation selectivity. Hence, the maximum output power density reached was 0.96 W m^−2^ at only 0.5 and 0.01 M NaCl solutions when tested in RED. 

Based on previous studies, it can be concluded that cellulose-based membranes can be proposed as alternative materials for RED. However these sustainable membranes must balance high ion selectivity and low electrical resistance, a compromise that is difficult to achieve due to the nature of cellulose and complex structure, which often involves chemical modification, the use of filler and reinforcement, and the necessity of steps for precise structural control. Moreover, the inherent high hydrophilicity arising from cellulose causes swelling in high salinity conditions, opening transport channels, reducing selectivity, and increasing electrolyte leakage, and this requires crosslinking that has be optimized to avoid the adverse effects on ionic conductivity. Furthermore, the organic nature of cellulose-based membranes makes them prone to fouling by organics, colloids, and scaling salts, as well as to concentration polarization, all of which reduce ionic flux and energy efficiency over time. Thus, introducing an antifouling agent to these membranes is highly essential. Membrane thickness is another limitation: thinner membranes reduce resistance but compromise structural integrity against RED stack compression, and thus, thickness needs to be optimized. From a practical viewpoint, many high-performance membrane designs that have been achieved rely on surface modification, crosslinking, or composite fabrication, which remain difficult and costly to scale with uniform quality. Finally, RED performance is system-level dependent, and the integration of cellulose-based membranes into optimized flow channels, spacer profiles, and turbulence-inducing profiles is still a technical challenge to ensure efficiency and durability in real-world applications. Table 9 presents a summary of previous studies of cellulose-based membranes for REDs. 

## 10. Challenges and Recommendations

Cellulose-derived IEMs and separators have significant sustainability advantages because they are renewable, inexpensive, and environmentally friendly. However, their use in electrochemical devices for power generation and storage, including fuel cells, VRFBs, water electrolysis, supercapacitors, and RED, encounter several persistent challenges stemming from the complex interdependence of essential performance parameters. One of the greatest challenges is maintaining ionic conductivity versus mechanical/chemical stability. Unmodified pure cellulose intrinsically possesses poor ion conductivity because of the absence of sufficient ion-percolating sites among its glucose units connected by β-1,4-glycosidic linkages. It typically requires chemical functionalization with ionic groups (e.g., -SO_3_H, -COOH, -PO_4_^3−^, and -OH) or in situ polymerization and grafting ion-conductive polymers (e.g., SPS, PSSA, SA, and SPES) to enable the transport of ions. Such chemical modifications, although increasing the density of ion-conducting channels, are likely to disrupt the ordered cellulose microstructure, leading to excessive swelling, dimensional instability, and a loss of mechanical integrity. Excessive modification aggravates these properties, while insufficient modification does not provide sufficient conductivity, and thus, the optimization of parameters is highly sought. Chemical stability in harsh operating environments is another vital concern. Exposure to aggressive oxidizing media (acidic or basic electrolytes) and heat causes degradation and reduces the lifetime of the membranes. Excessive hydration and swelling also undermine dimensional stability and lead to mechanical degradation. Susceptibility to biofouling in aqueous system separators, particularly in membrane water electrolyzers and RED, reduces operating efficiency and increases maintenance requirements. The majority of studies lack in situ (real-device) durability testing, which limits the deployment of various laboratories to industry. From a manufacturing perspective, scaling without compromising performance is economically and technically demanding. Multi-step fabrication techniques and intricate modification chemistries add cost, making commercial use in large-scale in renewable energy sector rather challenging for cellulose-based composite membranes. Addressing these challenges entails achieving a balance between inter-related properties. The main challenges and recommendations for various relevant electrochemical energy systems are presented in Table 10.

## 11. Concluding Remarks

Cellulose-based IEMs/separators for electrochemical energy systems were comprehensively reviewed. The use of various cellulosic forms was found to provide viable and green alternatives to conventional synthetic membranes/separators used in electrochemical energy conversion devices, including fuel cells (PEMFCs, DMFCs, AEMFCs, and MFC)s, water electrolyzers (PEMWE and AEMWE), LIBs, VRFBs, supercapacitors, and RED. Chemically modified cellulose such as CA, sulfonated cellulose, and physical forms such as BC, MCC, CNC, CNWs, and nanofibers were used as fillers or for reinforcement of the polymer matrix for the development of composite membranes with enhanced properties. The renewable origin, low toxicity, and structural flexibility of these membranes make them especially beneficial for future sustainable energy uses. However, to realize the full potential of these materials, it is imperative to overcome several key challenges including their intrinsic low ionic conductivity, limited chemical stability in harsh conditions (acidic or basic), and the challenges in applying the necessary chemical modifications. Advances during the past decade have focused on enhancing cellulose’s ionic transport properties using various strategies including functionalization, cross-linking and filler incorporation, and reinforcement for nanocomposite formation. Chemical modification strategies such as RIG, direct chemical treatment, blending, crosslinking, and incorporation of sulfonated GO, TiO_2_, or SiO_2_ have been employed. Despite the great potential shown by these approaches, the dilemma of achieving a balance between various physical and chemical properties persists. Therefore, developing new membranes requires harmonizing mechanical stability, ionic conductivity, and selectivity, maintaining chemical and thermal stability in corrosive electrolytes, limiting swelling in aqueous conditions, resisting fouling, overcoming the lack of in situ (real-device) durability testing, obtaining consistent pore and thickness control, performing early-stage integration into reasonable device models, and improving scalability aspects. The combination and prioritization of membrane properties are highly dependent on the application. For instance, fuel cells demand exceptional proton conductivity, chemical stability, and mechanical integrity under harsh operating conditions, whereas processes such as RED can tolerate comparatively moderate property thresholds, focusing instead on permselectivity and cost-effectiveness. In summary, sustained research and development efforts can advance cellulose-based conductive membranes toward achieving the requisite physicochemical and electrochemical properties and cost effectiveness for energy conversion and storage devices, thereby unlocking their full potential to transform sustainable and efficient energy technologies.

## Figures and Tables

**Table 1:** Methods for the fabrication of cellulose-based membranes for electrochemical applications.

Methods	Merits	Demerits	Applications
Solution Casting/ phase Inversion	- Simple and scalable - Good control over the thickness of membranes - Suitable for cellulose derivatives	- Use of toxic solvents - Limited porosity - Post-treatment is needed for stability	PEMs, AEMs, battery and supercapacitor separators
Electrospinning	- High surface area - Porous and flexible structures - Good ionic conductivity	- Requires high-voltage setup - Often involves toxic solvents - Post-regeneration is needed	Battery and supercapacitor separators, AEMs and PEMs
Regeneration from cellulose solutions	- Uses renewable cellulose - Environmentally friendly solvents can be used (e.g., ILs)	- Expensive or difficult-to-recycle solvents - Low mechanical strength	PEMs, AEMs, and separators
In-situ copolymerization/grafting	- Introduces functional groups to the cellulose backbone - Chemical bonding with cellulose backbone	- Complex process - Radiation sources or chemical initiators are required - Expensive in some cases	PEMs and AEMs for fuel cells and electrolyzers
Freeze-drying	- High porosity and surface area - Retains nanostructure integrity	- Energy-intensive - Poor mechanical strength unless reinforced	Supercapacitors, catalytic supports, and separators for VRFBs
Hot pressing/film casting of nanocellulose	- Solvent-free or low solvent use - Produces dense and robust films	- Long drying times - involve CNF/CNC fabrication, which is energy-intensive	PEMs and battery separators
Composite membrane formation	- Tailored properties - Combines the strengths of multiple components/fillers and substrates	- Interfacial compatibility issues - Potential phase separation - Complex fabrication	All electrochemical systems (PEMFCs, LIBs, VRFBs, etc.)

**Table 2:** Properties of various cellulose nanostructures for membrane applications.

Cellulose Structure	Merits	Demerits	Imparted Functions
BC	- Exceptional purity - High crystallinity - Robust nanofibrillar network	- Dense microstructure restricts ion diffusion - Limited large-scale production	- Provides mechanical stability - Enhances water uptake - Facilitates ion transport
CNCs/NCCs	- Rigid rod-like morphology - High surface area - Abundant reactive sites for functionalization	- High loading causes brittleness - Reduces processability of composites	- Improves stiffness and dimensional stability - Enables surface chemical modification
CNFs	- Flexible, entangled fibrillar network - Rich in OH^−^ - High aspect ratio	- High aqueous viscosity complicates processing - Strong tendency to aggregate	- Provides mechanical reinforcement - Offers tunable ionic conductivity - Enhances flexibility
CNWs	- Higher aspect ratio and crystallinity than CNCs - Strong reinforcing ability	- Aggregation and poor dispersion in polymer matrices - Processing challenges in blending	- Improves dimensional stability - Enhances proton conductivity when functionalized

**Table 3:** Summary of previous studies on cellulose-based membranes for PEMFCs.

Membrane Type	Modification	Membrane Thickness (µm)	Ion Conductivity (mS cm^−1^)	Power Density (mW cm^−2^)	Water Uptake (%)	Refs.
Nafion 115	NA	126	74	320	NA	[156,157]
Nafion 117	NA	178	13.3 (30 °C/100%RH)	30	35	[158]
Crosslinked Cell/SA	Chemical	NA	23 (25 °C)	NA	53	[116]
BC/AMPS	Chemical	NA	29	97	264	[120]
NCF/carboxylate & NCC/carboxylate	Chemical	32/ 30	0.05 at 100 °C & 4.6 at 120 °C	0.79 & 1.2	NA	[134]
CMBC/PANI	Chemical	NA	25.2	NA	NA	[124]
BC/PSSA	Chemical	NA	0.1 (94 °C)	NA	NA	[159]
BC/PSSA	Physical	100	NA	40.0	NA	[124]
BC/Nafion^®^	Physical	NA	140 (94 °C/98%RH)	NA	NA	[125]
Nata de Cassava Bacterial Cellulose	Chemical	0.4154	79.0 (80 °C)	25	90	[126]
CB/PMOEP	Chemical	42	0.1 (98% RH)	NA	206	[127]
BC/P(bisMEP)	Chemical	132	0.03 (80 °C/98%RH)	NA	NA	[128]
BC/Fuc	Physical	50–80	1.6 (94 °C/98%RH)	NA	NA	[129]
BNC/LS	Physical	85	23 (94 °C/98%RH)	NA	NA	[131]
BC/PANI-BG-BF4	Physical	20–25	0.052 (180 °C)	NA	NA	[130]
MCC-Im	Chemical	1150	0.000021 (70 °C)	NA	NA	[136]
1.7NCC-Im	Chemical	100	0.27 (140 °C)	NA	NA	[37]
1.3 NCC-Im	Chemical	234	4.0 (160 °C)	NA	NA	[38]
2.66 NCC-Tri	Chemical	150	0.001 (175 °C)	NA	NA	[139]
NCC/Tri	Physical	100–200	0.013 (120 °C)	NA	NA	[140]
CNF- COOH	Physical	500	0.00001 (60 °C)	NA	NA	[143]
NCC-5/COOH-10	Chemical	0.07	218 (90 °C)	NA	NA	[56]
CNF/COOH	Physical	15	1.0 (30 °C/75%RH)	NA	NA	[135]
P-CNF/SPES	Physical	85–120	154 (80 °C, 100% RH)	NA	45	[142]
NCC/SFPEAK	Physical	90–110	0.245 (90 °C)	NA	NA	[160]
s- PBI/cell/SiO_2_	Physical	45–55 × 10^3^	9.11 (20 °C)	NA	NA	[151]
S-PEEKK/Am3-sNCC	Chemical	NA	0.210 (100 °C)	NA	NA	[144]
CNC/SPEEK	Physical	70	0.186 (95 °C and 95% RH)	NA	NA	[143]
Cell/Nafion & Cell/RDP	Physical	196–206	NA	23 10	NA	[161]
S-CNFs	Physical	30	2.0 (120 °C)	4.1	NA	[162]
CA-rGO-PVDF	Physical	120	0.4	NA	NA	[147]
CA/GO	Physical	25	15.5	519	NA	[163]
Tempo-oxidized and sulfonated CNF	Chemical	50	1.76 (25 °C, 100% RH)	NA	63	[118]
CNF-g-PMMA/PF	Chemical	137	NA	542	NA	[121]
NCC/PVA/SGO	Physical	NA	11.0 (80 °C, 100% RH)	2.9	147	[153]
NCC/PVA	Physical	70	3.0	10.3	120	[154]
NCC-Im & NCF-Im	Physical	400 & 500	0.0326 & 0.021	NA	NA	[145]
MCC/RDP/PO_3_–H	Chemical	210	1.12	16 (in air) 34.3 (in oxygen)	NA	[155]
SEC/SPEEK	Physical	NA	109.2	NA	30	[152]

**Table 4:** Summary of previous studies on cellulose-based PEMs for DMFCs.

Membrane Type	Modification	Membrane Thickness (µm)	Ion Conductivity (mS cm^−1^)	Power Density (mW cm^−2^)	Water Uptake (%)	Methanol Permeability (μcm^2^ s^−1^)	Selectivity (kS s cm^−3^)	Ref.
Nafion 117	NA	178	15	126.04	30	0.884	NA	[175]
AMPS -BC	Chemical	NA	29.0	61.0	275	0.564	NA	[120]
PVA/CS/CNC-HNO_3_	Physical	NA	64.2	NA	78	0.031	18.1	[166]
Nafion/5%-CW	Physical	NA	17.0(70 °C)	91.0	29	2,331,070	400	[65]
CNF/SPES	Physical	110–130	0.13	NA	NA	0.445	89	[150]
CNF/SPES/FAA-20	Physical	60–100	0.264(80 °C)	87.22	NA	NA	NA	[144]
Cell/PTA/Im	Physical	500	0.214	NA	38.68	0.0214	0.00004838	[169]
NC/Im/m-PTA5	Physical	500	31.9	NA	60.3	0.0174	NA	[170]
NC-10SSA	Physical	20	3.2	NA	60	NA	NA	[171]
SPSF/CW-Ser	Physical	NA	0.234 (80 °C)	73.8	50	0.76	NA	[146]
CNF/UiO-66-NH_2_/SPS	Physical	80–100	0.20 (80 °C)	NA	NA	0.55	NA	[167]
CNC/PVA	Physical	NA	3.0	10.3	NA	NA	NA	[154]
MCC/DAC/SPEEK	Physical	NA	0.1	NA	522	NA	NA	[168]
CA-g-PSSA	Chemical	30	4.77	24.6	31.4	0.55	8.65	[176]
CA-g-PPVA	Physical	1200	0.035	NA	68.7	0.000108	NA	[177]
CNF-SSASPEI	Physical	NA	16.7(80 °C)	4.32	NA	0.0822	NA	[172]
SPS/CNDs	Chemical	130	35.5	NA	45.6	3.5	0.00101	[173]

**Table 5:** Summary of previous studies on cellulose-based membranes for AEMFCs.

Membrane Type	Modification	Membrane Thickness (µm)	Ion Conductivity (mScm^−1^)	Power Density (mWm^−1^)	Water Uptake (%)	Ref.
BC/TiO_2_/CHPTAC	Physical	NA	93.0 (80 °C)	NA	114	[181]
BC-PDDA-OH-	Physical	NA	51.0	NA	NA	[182]
BC/PMACC	Chemical	NA	10.0 (94 °C, 98%RH)	NA	1549	[183]
BC/LDH	Chemical	NA	70.7 (80 °C)	13.6	NA	[184]
BC/TiO_2_/VBTAC	Chemical	NA	100.5(80 °C)	40.2	55	[62]
Crosslinked DABCO–CNF/DABCO–PS	Chemical	NA	74.0(25 °C)	NA	59.5	[187]
QPPO/QCNC	Chemical	20	60.0 (80 °C)	392.0	17	[185]
QPPO/QCNF/QGO	Physical	NA	114.0 (80 °C)	NA	89.0	[186]
CA-NF/CSSIPN	Physical	150	21.0	NA	NA	[186]
Crosslinked QPVA/QNC	Physical	100	15.0	1.95	230	[190]
PBC3/QPPO	Chemical	NA	62.6	0.43	NA	[191]

**Table 6:** Summary of previous research studies on cellulose-based membranes for LIBs.

Membrane Type	Modification	Membrane Thickness (µm)	Ion Conductivity (mScm^−1^)	Thermal Shrinkage (% at °C)	Mechanical Strength Stress (MPa)/ Strain (%)	Capacity Retention (% of the Number of Cycles)	Ref.
PE	NA	20	0.65	90 at 150	12.5/17	96 at 100	[3]
PP (Celgard 2400)		25	0.38	99 at 200	15.3/12	82 at 100	[18]
PP-PE-PP	NA	25	0.18	99 at 200	90/55	55 at 200	[179]
PP-PE-PP (Celgard 2340)	NA	38	3.39	34 at 200	140/47	82 at 50	[182]
BC	Physical	13	NA	0 at 180	78/5.0	NA	[177]
OBCS-200	Chemical	57	2.9	0 at 160	9.9/0.55	90 at 100	[180]
PPy/BC	Chemical	25	1.28	0 at 200	22.1/4.0	76 at 200	[179]
BC-Al_2_O_3_	Physical	30	4.91	0 at 200	122/0.3	89 at 50	[182]
BCZC	Physical	25	2.14	3 at 200	18.2/10.0	98 at 100	[3]
BCNCs/PEBAX	Physical	35	9.79	0 at 150	14.9/55.2	NA	[181]
BZP	Chemical	52	1.15	0 at 180	NA	86 at 1600	[178]
ZIF-8@BC-2	Chemical	NA	1.12	0 at 200	25.6/4.0	89 at 100	[183]
ANFs/BC	Physical	30	12.5	0 at 160	NA	93 at 100	[184]
BC/HNTs	Physical	30	5.13	0 at 200	NA	95 at 100	[185]
TOBC	Chemical	30	13.45	0 at 200	110/9.5	94 at 100	[186]
PDA/BC	Chemical	NA	1.89	0 at 180	NA	NA	[187]
CPC	Chemical	20	0.22	0 at 200	NA	98 at 65	[197]
PPNBs/CS	Physical	20	1.04	30 at 260	50/4.0	92 at 150	[198]
CNCs/PAN	Physical	30	2.82	7.3 at 200	22.5/11.0	97 at 100	[189]
mCNC	Chemical	75	2.00	NA	NA	93 at 100	[188]
MCNC	Physical	150	2.7	0 at 100	55/4.0	90 at 60	[53]
PVdF/NCC	Physical	20	1.45	NA	99/2.0	NA	[43]
CNP	Physical	19	0.77	0 at 150	21/NA	87 at 100	[191]
ECM	Physical	12	0.26	0 at 160	60/5.0	78 at 100	[192]
CNF	Physical	NA	1.90	0 at 160	68/11.00	97.5 at 100	[193]

**Table 7:** Summary of previous studies on cellulose-based membranes for VRFBs.

Membrane Type	Modification	Membrane Thickness (µm)	Proton Conductivity (mS cm^−1^) Vanadium Permeability (×10^−7^ cm^2^ min^−1^)	EE (%)/Number of Cycles at Current Density (mA cm^−2^)	Mechanical Strength Stress (MPa)/ Strain (%)	Ref.
Nafion 115	NA	125	15.0/NA	NA	20/155	[208]
Nafion 115	NA	125	6.0/NA	NA	35/238	[209]
Nafion 212	NA	50	NA/54	NA	NA	[211]
Nafion 212	NA	50	71.6/3.2	82/100 at 80	NA	[213]
Nafion 212	NA	60	44.0/4.4	NA	10/89	[214]
Nafion/PBI	Physical	25	111.0/0.0195	85/300 at 60	NA	[234]
Nafion/PBI	Chemical	51	32.3/13.3	81.3/300 at 200	32/81	[235]
CNC/PVDF-HNF	Physical	77	16.0/NA	88.2/650 at 100	65/10	[208]
BC/PVDF-HNF	Physical	200	9.5/NA	79.5/300 at 100	114/14	[209]
CNC/PVDF-HNF	Physical	20	6.9/NA	NA	48/4.5	[210]
SCNC/MXene/ PVDF-HFP	Physical	40	15.8/660	90.7/120	52/0.8	[11]
Nafion-PDDA/CNC	Physical	51	NA	89/50 at 60	NA	[212]
BC-PFSA	Physical	NA	60.5/8.6	78.9/100 at 80	NA	[213]
CNC-SPES	Physical	70	29.1/3.7	82/200 at 100	2/25	[214]

**Table 8:** Summary of previous studies on cellulose-based membranes for application in supercapacitors.

Membrane Type	Modification	Membrane Thickness (µm)	Ionic Conductivity (S cm^−1^)	Power Density (W m^−2^)	Ref.
Ppy/CNT/BC	Chemical	0.12	21	228	[250]
ZIF-67-Ppy/PDA/BC	Chemical	70,000	NA	209.09	[251]
BC/GN/Ppy	Chemical	70	621	444	[252]
C-pHEMA/C-p-cellulose	Chemical	100	0.224	124	[253]
GO/CNC	Chemical	NA	64.7	123.2	[254]
(PANI@CNT−CNC/PVA− PAA)	Chemical	40	0.0044	164.6	[255]
CNF	Physical	25	0.00269	124.76	[256]
CTOCN/MnO_2_	Chemical	0.025	NA	171.1	[257]
CF-CNF/MWCNT-Has	Physical	228.6	NA	174.5	[258]
CNF@Ni-HITP	Physical	350	103	103	[259]
CNF/VGCF/Ppy	Chemical	NA	11.25	678.66	[261]
CNFs/rGO/PPy	Chemical	NA	2.5	405	[262]
CNF/CNT/Ppy	Chemical	NA	NA	200.8	[263]
Cellulose/MWCNT/rGO/Co_3_O_4_/SnO_2_	Chemical	NA	NA	215	[264]
PDADMAC/CNF	Physical	30	0.005	185.3	[115]
(LS/PPy)/LCNFs	Physical	225	160	197	[265]
LiTFS–LiSMC	Chemical	500	0.001	74.11	[266]
MC/CuS@rGO	Physical	70	1.07 × 10^−7^	NA	[267]
CMCM	Physical	NA	NA	210	[268]
CMC/PEDOT: PSS	Physical	98	45.1	116.4	[269]
[PEDOT/Al_2_O_3/_NaCMC] PHMeDOT	Chemical	NA	NA	0.0006	[270]
Glycerol NaCMC-PC- LiClO_4_	Physical	310	0.000355	6.18	[271]
Cellulose/RGO/silver/Fe_2_O_3_	Chemical	620	NA	NA	[272]
Cell/PDA/PPy	Chemical	134	0.59	32	[273]
NC/RGO/PANI	Chemical	40	NA	79.71	[274]
NPC	Chemical	NA	NA	52.74	[275]
PC	Chemical	NA	0.606	255	[276]
A520-SC	Chemical	NA	0.172	135.14	[277]
Cell/PEDOT:PSS	Chemical	NA	0.77	NA	[278]
CFA	Physical	NA	NA	409	[279]
FeOCI@CDCA	Physical	NA	0.26	647	[280]
ASC	Chemical	NA	0.265	64.83	[281]
Cellulose	Chemical	150	0.2986	130	[282]
NSHPAs	Physical	500	NA	329	[283]
RC @PVDF	Physical	20	3.87 × 10^−6^	173	[114]
CCM	Physical	15	0.01632	145.6	[284]
CWCNF	Physical	25	0.003	1768	[285]

**Table 9:** Previous studies on cellulose-based membranes for REDs.

Membrane Type	Modification	Membrane Thickness (µm)	Ion Conductivity (mS cm^−1^)	Power Density (W m^−2^)	Ref.
NBC & PBC	Physical	90	1.00 & 0.42	0.23	[289]
BC-CMC & BC-HACC	Chemical	20	0.78	2.25 (+ve) & 0.42 (−ve)	[290]
NBC/NGO & PBC/PLDH	Physical	7	0.52–0.64	0.7	[291]
NBC/PSS & PBC/PDA	Chemical	160	1.40 & 0.8 × 10^−3^	0.99 & 1.14	[292]
MXene/CNF	Physical	46	4.00	0.15	[293]
GO/CNFs	Physical	9	135	4.19	[294]
g-C_3_N_4_/CNF	Physical	50	14	0.15	[295]
CNF/CNTs	Physical	6.76	NA	4.67	[296]
CNF/2D-GO	Physical	6	0.20	12.04	[297]
CNF/BTCA/SHP	Physical	20	8.10	NA	[298]
T-CNF & E-CNF	Physical	40	17 & 16	2.87	[299]
Bamboo-CNF	Physical	30	0.36	2.2	[33]
C-CNF	Chemical	50	NA	10.1	[300]
P-Wood & N-Wood	Physical	1000	0.20 & 0.40	0.00514	[301]
TOCNs/PET	Physical	21	NA	0.96	[302]

**Table 10:** Summary of challenges, recommendations, and target applications of cellulose-based IEMs/separators for electrochemical energy systems.

Challenges	Recommendations	Target Applications
Performance trade-off between ionic conductivity, selectivity, barrier properties and mechanical strength	- Apply hybrid (composite) designs with optimum combination of CNF with polymer matrices/inorganic fillers - Adopt fabrication methods to ensure balance conductivity, selectivity, barrier property and mechanical integrity	Various fuel cell types, VRFBs, PEMWE, AEMWE and RED
Chemical and thermal degradation in harsh electrolytes (acidic, alkaline, oxidative/reductive)	- Employ stable cellulose derivatives (e.g., cellulose acetate, carboxymethylated cellulose, etc.) - Use CNWs or CNF matrices such as PPO- Reinforcement with oxidation-resistant fillers (Mxene, GO, SiO_2_, etc.).	All systems
Lack of sufficient in-situ durability tests in real devices	- Conduct accelerated aging and realistic cycling tests- Integrate membranes into device prototypes at early stage	All systems
Complex, energy-intensive, and low-throughput fabrication processes	Develop roll-to-roll or extrusion manufacturing facilities, optimize solvent recovery, use greener nanocellulose production with reduced energy demand	All systems
Inconsistent pore size and thickness control at scale	Apply scalable templating/phase-separation methods, integrate in-line monitoring for porosity and thickness uniformity during production	All systems
Lack of optimization studies on fabrication parameters	- Apply optimization techniques to obtain the best combinations allowing development of desired structures- Use DFT and AI tools to design robust membranes- Apply optimization techniques for investigating operating parameters to obtain maximum performance	All system

## Data Availability

No new data were created or analyzed in this study. Data sharing is not applicable to this article.

## Abbreviations

AEM	Anion exchange membrane
AEMFC	Anion exchange membrane fuel cell
AEMWE	AEM water electrolyzer
ASC	All-nanofiber asymmetric supercapacitor
ATRP	Atom-transfer radical polymerization
BC	Bacterial cellulose
BC-CMC	Negatively charged carbomethyl bacterial cellulose membranes
BC-HACC	Positively charged chitosan quaternary ammonium bacterial cellulose membrane
BNC	Bacterial nanocellulose
BTCA CA	Butane 1,2,3,4 tetracarboxylic acid Cellulose acetate
CCM	Cotton cellulose membrane
CDCA	Cellulose-derived carbon aerogel
CE	Coulombic efficiency
CF	Carbon fiber
CFA	Carbon fiber aerogel
CMC	Carboxymethyl cellulose
CNCs	Cellulose nanocrystals
CNFs	Cellulose nanofibrils
CNTs	Carbon Nanotubes
CNW	Cellulose nano-whisker
CQD	Carbon quantum dot
CS	Chitosan
DMAC	Dimethylacetamide
DMFC	Direct methanol fuel cell
EDLC	Electric double-layer capacitors
EE	Energy efficiency
EES	Electrochemical energy storage system
EG	Ethylene glycol
GN	Graphene
GO HPMC	Graphene oxide Hydroxypropyl methyl cellulose
ICM	Ion conducting membrane
IEM	Ion exchange membrane
IPA	Isopropyl alcohol
LCNFs	Lignin-containing Cellulose Nanofibrils
LDH	Layered double hydroxide
LIBs	Lithium-ion batteries
LISBs	Lithium-ion sulfate batteries
LiTFS–LiSMC	Lithium tri-fluoromethanesulfonate-methyl cellulose
LS	Lignosulfonates
LS	Lignosulphonate
MBAA	N N′-methylenebisacrylamide
MC	Methylcellulose
MCC	Microcrystalline cellulose
MFC	Microbial fuel cell
MMA	Methyl methacrylate
MOF	Metal organic frameworks
MWCNT	Multi wall carbon nanotube
NaCMC	Sodium caboxymethylcellulose
NBC	Negatively charged bacterial cellulose
NCC	Nanocrystalline cellulose
NMMO	N-methylmorpholine *n*-oxide
NSHPAs	N, S dual-doped hierarchical porous carbon aerogels
PAA	Poly(acrylic acid)
PAEK	Poly(aryl ether ketone)
PANI	Polyaniline
PBC	Positively charged bacterial cellulose
PBI	Polybenzimidazole
PDA	Polydopamine
PDADMAC	Poly (diallyldimethylammonium chloride)
PEDOT	Poly(3,4-ethylenedioxythiophene)
PEEK	Poly(ether ether ketone)
PEM	Polymer electrolyte membranes
PEMFC	Proton exchange membrane fuel cell
PEMWE	Poron exchange membrane water electrolyzer
PEO	Poly(ethylene oxide)
PET	Polyethylene terephthalate
PF	Phenol formaldehyde
PFSA	Perfluorosulfonic acid
pHEMA	Poly(2-Hydroxyethyl Methacrylate)
PMACC	Poly(methacroylcholine chloride) (pmacc)
PMOEP	Poly(methacryloyloxyethyl phosphate)
PNIMPAM	Poly(N-isopropylacrylamide)
Ppy	Polypyrrole
PRO	Pressure retarded osmosis
PSS	Polystyrene sulfonate
PSSA	4-polystyrene sulfonic acid
PTA	Phosphotungstic acid
PVA	Polyvinyl alcohol
PVDF	Poly(vynilidene fluoride)
QPPO	Quaternized poly(phenylene oxide)
RDP	Bis(diphenyl phosphate)
RED	Reverse electrodialysis
RFB	Redox flow battery
rGO	Reduced graphene oxide
RH	Relative humidity
SA	Sulfosuccinic acid
SA	Sulfonic acid
SEC	Sulfo ethyl cellulose
SHP	Sodium hypophosphite
SiO_2_	Silicone dioxide
SPEEK	Sulfonated poly(ether ether ketone)
SPEI SPSF	Sulfonated poly(ether imide) Sulfonated polysulfone
SPES	Sulfonated poly (ether sulfone)
TiO_2_	Titanium dioxide
TOCNF	TEMPO-oxidized cellulose nanofibers
VE	Voltage efficiency
VGCF	Vapor hrown carbon fiber
VRFB	Vanadium redox flow battery
ZIF-67	Zeolitic imidazolate framework
